# Automatic discovery of image-based signatures for ipilimumab response prediction in malignant melanoma

**DOI:** 10.1038/s41598-019-43525-8

**Published:** 2019-05-15

**Authors:** Nathalie Harder, Ralf Schönmeyer, Katharina Nekolla, Armin Meier, Nicolas Brieu, Carolina Vanegas, Gabriele Madonna, Mariaelena Capone, Gerardo Botti, Paolo A. Ascierto, Günter Schmidt

**Affiliations:** 1 Definiens AG, Munich, Germany; 2 Istituto Nazionale Tumori IRCCS Fondazione G. Pascale, Naples, Italy; 3 Department of Translational Medical Sciences and Center for Basic and Clinical Immunology Research (CISI), University of Naples Federico II, Naples, Italy

**Keywords:** Cancer immunotherapy, Melanoma, Computer science, Predictive markers

## Abstract

In the context of precision medicine with immunotherapies there is an increasing need for companion diagnostic tests to identify potential therapy responders and avoid treatment coming along with severe adverse events for non-responders. Here, we present a retrospective case study to discover image-based signatures for developing a potential companion diagnostic test for ipilimumab (IPI) in malignant melanoma. Signature discovery is based on digital pathology and fully automatic quantitative image analysis using virtual multiplexing as well as machine learning and deep learning on whole-slide images. We systematically correlated the patient outcome data with potentially relevant local image features using a Tissue Phenomics approach with a sound cross validation procedure for reliable performance evaluation. Besides uni-variate models we also studied combinations of signatures in several multi-variate models. The most robust and best performing model was a decision tree model based on relative densities of CD8+ tumor infiltrating lymphocytes in the intra-tumoral infiltration region. Our results are well in agreement with observations described in previously published studies regarding the predictive value of the immune contexture, and thus, provide predictive potential for future development of a companion diagnostic test.

## Introduction

Checkpoint blockade immunotherapies are very successful for some cancer patients, however, a large number of patients do not benefit from these relatively costly therapies, but on the contrary suffer from severe adverse events. Therefore, potential biomarkers for companion diagnostic tests are being actively studied which will allow predicting a patient’s individual likelihood of clinical therapy response. In particular, with regard to combination therapies where multiple immunotherapies (e.g., ipilimumab and nivolumab)^1^, or conventional therapies and immuno-therapies are combined, such tests are becoming increasingly important due to the large number of possible combinations available and the increased frequency and severity of adverse events in combination therapies. For immunotherapies based on immune checkpoint inhibitors in the context of malignant melanoma different biomarker strategies are being investigated. Those include, for example, the PD-L1 tumor status, tumor-infiltrating lymphocytes (TILs), mutational and neoantigen burden, immune-related gene signatures as well as multiplexed immuno-histochemistry^2–5^. However, for the anti-CTLA-4 checkpoint inhibitor ipilimumab to date there are still no confirmed biomarkers available for predicting clinical therapy response while several candidates have been suggested. Peripheral blood cells or serum factors have been investigated as potential predictive markers in Ascierto *et al*.^6^, while the importance of the immune contexture was pointed out. Hamid *et al*.^7^ evaluated TILs, FoxP3 and IDO based on immunohistochemistry in tissue samples from patients treated with ipilimumab, and demonstrated that an increase of TILs during treatment is associated with clinical response to ipilimumab. In Ji *et al*.^8^ a high baseline expression level of immune-related genes in the tumor microenvironment was reported to be predictive for therapy response. Snyder *et al*.^9^ found that a high mutational load of the tumor is associated with therapy response based on whole-exome sequencing. In general, most data collected so far indicate that an immunologically more active tumor microenvironment is beneficial for response (“hot” tumors). Thus, systematically studying the immune cell infiltration at tumor sites using immunohistochemistry (IHC) may help in identifying strong and robust predictive biomarkers.

The availability of digital pathology and the advance of large-scale quantitative image analysis approaches for whole-slide images enable the systematic search for novel tissue- and immunohistochemistry-based biomarkers to predict therapy response and overall survival. In this retrospective study, we performed such a systematic search on multiplexed digital tissue images of malignant melanomas to identify signatures providing predictive value. In particular, we studied the immune contexture of the tumor and surrounding tissue aiming to reproduce an immune status-based signature in malignant melanoma similar to the Immunoscore^10^ developed for stage III colorectal cancer as addressed, e.g., by Capone *et al*.^11^. The study presented here was designed as a proof-of-concept study in conjunction with the larger MISIPI study^12,13^ with the goal to discover predictive biomarkers for ipilimumab response. In the MISIPI study an association between ipilimumab therapy response and the coexistence of low densities of CD8 + PD-L1− and high densities of CD163 + PD-L1+ cells in the invasive margin was found^14^. There, immune cell densities were considered in the invasive margin as well as in the center of the tumor. In our work, we automatically extracted a larger variety of image-based features emphasizing the feature locality by further subdividing the tumor microenvironment into compartments. Besides immune cell densities we investigated average distances between different immune cell types as well as relative densities, providing a more elaborate description of the immune status. To select the most stable and robust predictive features we first compared different feature selection methods with respect to their robustness for the given data set using Monte Carlo cross validation. After selecting the best uni-variate models based on the best feature selection method, a systematic comparison of different multi-variate models regarding their predictive potential was performed. Finally, we obtained the most robust multi-variate model given our data, and trained the final model for ipilimumab response prediction. Our approach is based on advanced image analysis including high precision multi-resolution co-registration, machine learning-based parameter-free cell segmentation and classification, as well as deep learning-based region classification for accurate differentiation between melanin and IHC marker-positive immune cells.

Large-scale systematic feature extraction, feature selection, and feature combination together with automatic image analysis as applied here, are core building blocks of the Tissue Phenomics approach which has been shown to be successful in prognostic biomarker discovery in other cancer types in previous work^15,16^.

## Materials and Methods

### Patient cohort

The patient cohort used in this study was collected in conjunction with the MISIPI study^12^ with 31 patients (10 female, 21 male, age 29–89 y) suffering from stage IV malignant melanoma. All patients were treated with up to four doses of ipilimumab and 12 patients showed ipilimumab therapy response (complete or partial response). 24 out of the considered 31 patients received ipilimumab as a second line therapy (mostly pretreated with dacarbazine or cisplatin and temodal), while for 4 patients ipilimumab was the third line of therapy. No patient had received more than two previous therapies. The follow-up period varied between 1 and 59.5 months (median 20.2 months) with a median overall survival time (median OS) of 23.1 months for all patients, 10.2 months for non-responders and median OS not reached for responders. Table 1 provides an overview of the patient cohort characteristics, Table 2 gives the results of a uni-variate and a multi-variate Cox regression analysis. For Kaplan-Meier plots of the stratification into ipilimumab responders and non-responders, as well as all response categories see Supplemental Fig. S1.

**Table 1:** Characteristics of the patient cohort.

**Total number** of patients	**31**											
**Patient age** Number of patients	**29**–**49** y 4				**50**–**69** y 14				**70**–**89** y 13			
**Gender** Number of patients	**Male** 21						**Female** 10					
**Median overall survival**	**All** 23.1 months				**Response** Not reached				**Non-response** 10.2 months			
**Follow-up period**	1–59.5 months											
	**Response**						**Non-response**					
**IPI response** Number of patients	**CR** 11			**PR** 1			**SD** 4			**PD** 15		
**Number of IPI doses** Number of patients	**1** 3			**2** 5			**3** 3			**4** 20		
Line of therapy at time of IPI	**Second line**						**Third line**					
**Previous therapies** Number of patients	Cisplatin + Temodal 9	Dacarbazine 10	Vemurafenib 2	Bleomicina 1	Fotemustine 1	Prame vaccine 1	Dacarbazine + CBDCA, Fotemustina 1		Temozolamide, Dabrafenib 1	CDDP + Temodal, Dabrafenib 1	Alovectina, Vemurafenib 1	NA 3
**BRAF mutation status** Number of patients	**Wild type** 22						**Mutated** 9					
**Metastasis category** Number of patients	**M1a** 10			**M1b** 7			**M1c** 7			**M1d** 7		
**LDH level** Number of patients	**Normal** 10				**Elevated** 12				**NA** 9			

CR: complete response, PR: partial response, SD: stable disease, PD: progressing disease. Metastasis categories: M1a: Soft tissue, M1b: lung, M1c: visceral, M1d: CNS. (NA: Data not available).

**Table 2:** Result of uni-variate and multi-variate Cox regression analyses on clinical features (total number of patients = 31, number of events = 17).

	n	Uni-variate			Multi-variate		
		HR	95% CI	p-value	HR	95% CI	p-value
**Previous therapies**							
Cisplatin + temodal	9						
Dacarbazine	10	0.832	0.253–2.733	0.762	1.691	0.132–21.68	0.686
Other previous therapies	9	0.917	0.280–3.008	0.886	1.008	0.075–13.50	0.995
**LDH level**							
Elevated	12						
Normal	10	0.237	0.064–0.883	**0**.**032**	0.404	0.068–2.416	0.321
**Metastasis category**							
M1a	10						
M1b	7	1.436	0.385–5.362	0.590	1.611	0.085–30.56	0.751
M1c	7	1.005	0.184–5.503	0.995	0.841	0.071–10.03	0.891
M1d	7	2.837	0.754–10.67	0.123	16.11	0.452–574.8	0.127
**Age**	31	0.985	0.952–1.019	0.373	0.989	0.938–1.042	0.672
**Gender**							
Female	10						
Male	21	1.904	0.619–5.857	0.261	1.012	0.102–10.06	0.992
**BRAF status**							
Mutated	9						
Wild type	22	0.560	0.205–1.533	0.259	0.615	0.097–3.922	0.607
**IPI response**							
Non-response	19						
Response	12	0.074	0.016–0.346	**9**.**3·10**^**−4**^			

For the multi-variate Cox regression the number of patients was lower due to missing data (number of patients = 19, number of events = 11). Metastasis categories: M1a: Soft tissue, M1b: lung, M1c: visceral, M1d: CNS.

### Experimental setup

Malignant melanoma tissue used in this study was collected at National Cancer Institute “G. Pascale” of Napoli, Italy. FFPE (formalin-fixed, paraffin-embedded) tissue blocks of melanoma biopsies performed (from 2007 to 2012) were retrieved from the Pathology Department archives. 31 FFPE samples were collected from 31 patients with metastatic melanoma, all of whom were subsequently treated with ipilimumab. 3-μm-thick serial tissue sections of FFPE blocks were cut for Haematoxylin & Eosin (H&E) and Immunohistochemical staining using antibodies specific for CD3, CD8, and FoxP3. For CD3, sections were stained using anti-CD3 rabbit monoclonal primary antibody (dilution 1:150) clone 2GV6 from Ventana Medical Systems Inc. & Spring Biosciences and the goat anti-rabbit + HRP visualization reagent from Ventana Medical Systems Inc. & Spring Biosciences (color brown was developed using 3,3-diaminobenzidine tetrahydrochloride DAB from Ventana Medical Systems Inc. & Spring Biosciences). For CD8, sections were stained using anti-CD8 monoclonal mouse primary antibody (dilution 1:100) clone C8/144B from Dako and the goat anti-mouse + HRP visualization reagent from Ventana Medical Systems Inc. & Spring Biosciences (color brown was developed using 3,3-diaminobenzidine tetrahydrochloride DAB from Ventana Medical Systems Inc. & Spring Biosciences). For FoxP3, sections were stained using anti-FoxP3 rabbit monoclonal primary antibody (dilution 1:500) clone SP97 from Ventana Medical Systems Inc. & Spring Biosciences and the goat anti-rabbit + HRP visualization reagent from Ventana Medical Systems Inc. & Spring Biosciences (color red was developed using Vulcan Fast Red from Ventana Medical Systems Inc. & Spring Biosciences). Each case was stained using a fully-automated staining protocol on a VENTANA BenchMark XT instrument with one hour incubation of primary antibody, following manufactured instructions. The specificity of marker expression and a re-review of the tumor histology were independently performed by two pathologists with extensive experience. All sections were imaged using a ZEISS Axio Scan.Z1 tissue scanner (Carl Zeiss, Jena, Germany) with a 20x objective (resolution 0.22 μm/pixel). Per patient four digital RGB whole-slide images were acquired with around 100 k × 100 k pixels (i.e. 1–2 GB per image after compression).

In previous work^17^, we found that the FoxP3 staining in this data set did not provide sufficient value for ipilimumab response prediction since only a very small number of positive cells were observed in general. Consequently, we did not include the FoxP3 readouts into our analysis but used the FoxP3-stained sections to identify regions of high melanin density (see section *Cell nucleus detection and segmentation*).

### The analysis workflow

**Figure 1:**
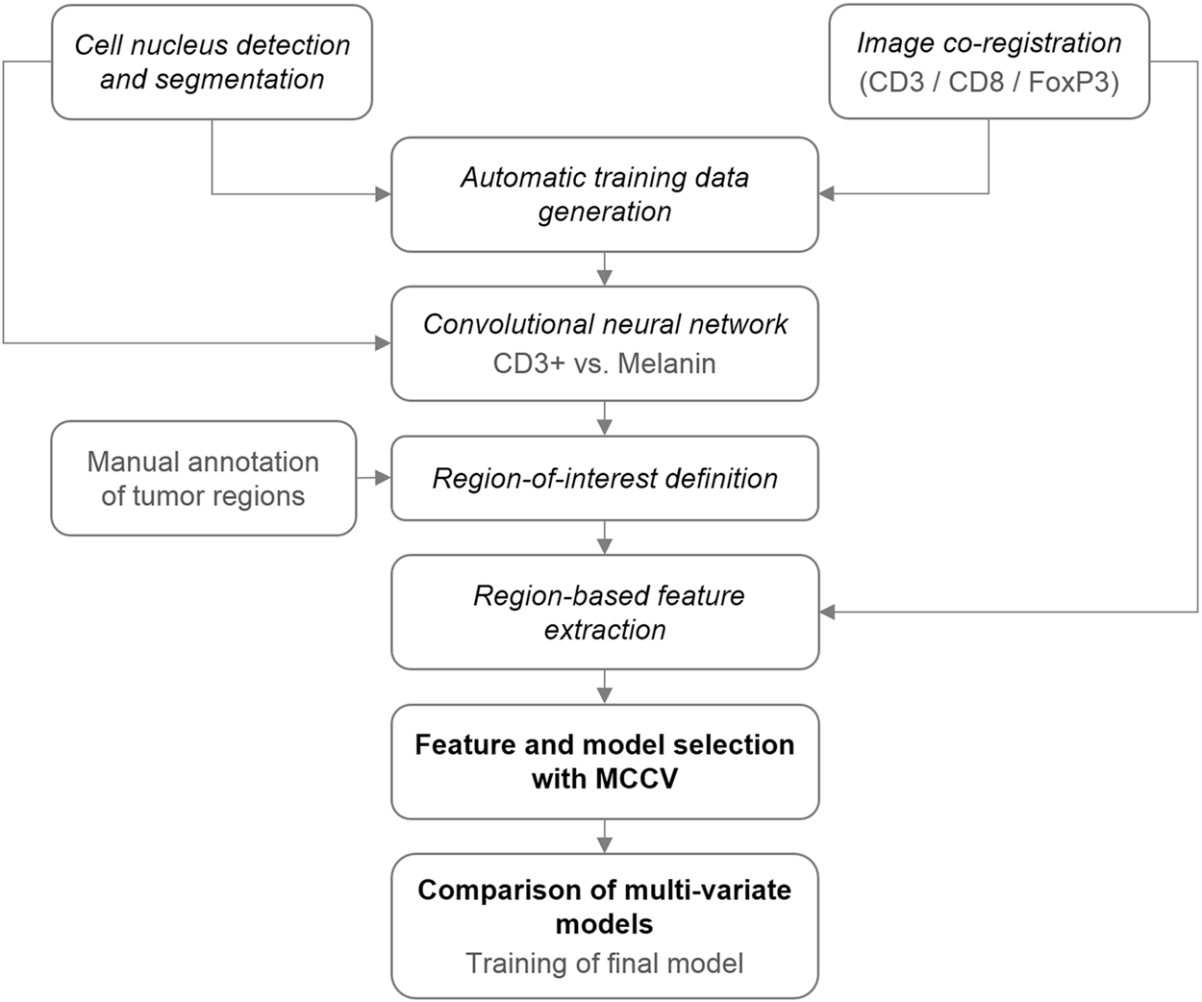
Overview of the processing workflow with the respective section names in the manuscript.

A general overview of the workflow to identify image-based signatures for ipilimumab response prediction is given in Fig. 1. First, cell and cell nucleus segmentation was performed on all images and all consecutive tissue section images per patient were co-registered. Both, the segmentation and co-registration results were then used in the next step for automatic generation of training patches to enable region-based classification of CD3+ and CD8+ cells versus melanin. Since CD3+ and CD8+ cells as well as melanin appear as brown roundish objects in the digital images, this deep learning-based classification step was introduced to robustly identify the objects-of-interest (i.e. CD3+, CD8+) and avoid false positive detections caused by melanin. Once all objects-of-interest were identified we defined regions-of-interest (ROIs) on the tissue sections using manual annotations of the tumor regions and exclusion areas. Based on the ROIs (tumor, compartments of the tumor microenvironment, stroma) local image features were extracted, taking into account objects-of-interest from different co-registered tissue sections. Next, the resulting set of image-based features was mined, including identification of a relevant feature subset and selection of the best stratification model using a Monte Carlo cross validation (MCCV) framework. Finally, the identified model along with the selected feature set was used to train the final model on the whole data set for application to new data.

### Basic image analysis

The first part of our analysis workflow involved the segmentation of stain-positive and stain-negative cell nuclei which was used as a basic building block in subsequent processing steps. In addition, a co-registration of the consecutive slices was performed to enable joint evaluation of immunohistochemically differently stained objects-of-interest in different physical tissue sections in one common coordinate system.

#### Cell nucleus detection and segmentation

**Figure 2:**
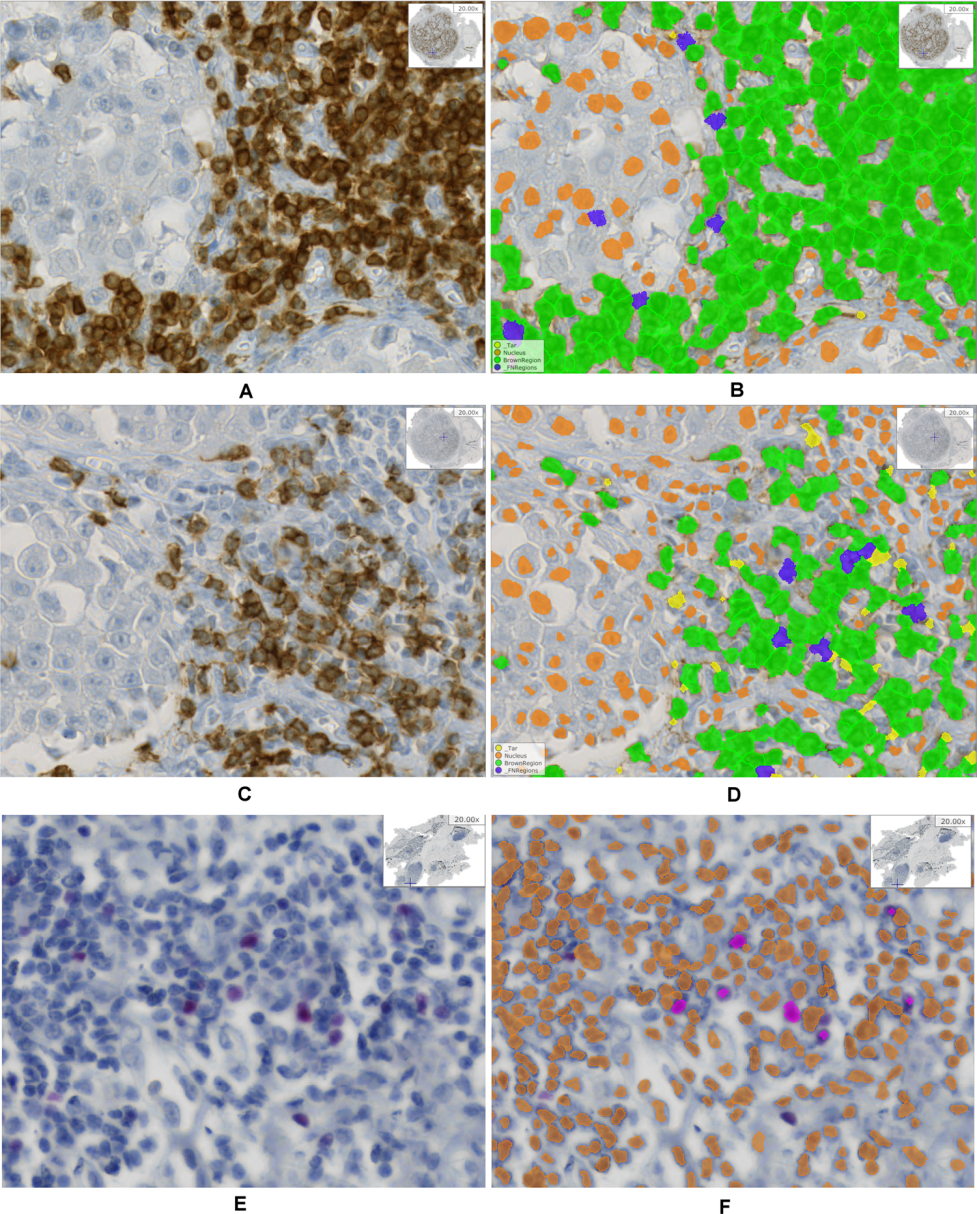
Example result of the cell nucleus segmentation. (**A**) CD3 raw image, (**B**) CD3 segmentation result, (**C**) CD8 raw image, (**D**) CD8 segmentation result, (**E**) FoxP3 raw image, (**F**) FoxP3 segmentation result. (**B**, **D**, **F**) Orange: IHC stain negative, green: CD3 or CD8 positive, magenta: FoxP3 positive, blue and yellow: artifacts.

Cell nucleus detection and segmentation was performed on all IHC stains, i.e. CD3, CD8, and FoxP3. To this end, we used a learning-based annotation-free approach^18^, which extracts clear and easy to identify representatives of cell nuclei based on domain knowledge regarding the shape consistency across representative tiles of multiple images, and trains a visual context model on this automatically extracted training data. The trained model is then applied to the whole-slide images to identify all nuclei including nuclei which are not easily detectable. As visual context model a random forest classifier in combination with long-range features^19^ is used. The resulting posterior probability maps are further processed using the same assumptions on shape consistency as used for initial object extraction to obtain a single-cell segmentation. Specifying the type of staining in the learning approach (e.g., nuclear stain, membrane stain, etc.) as well as the expected color of marker-positive cells or cell nuclei enables additional learning of IHC-positive cells versus IHC-negative cells on top of the cell segmentation. Thus, the approach performs both segmentation and classification of cells in IHC-positive and negative cells. For qualitative result examples see Fig. 2, for more details on the segmentation approach we refer to Brieu *et al*.^18^.

#### Image co-registration

**Figure 3:**
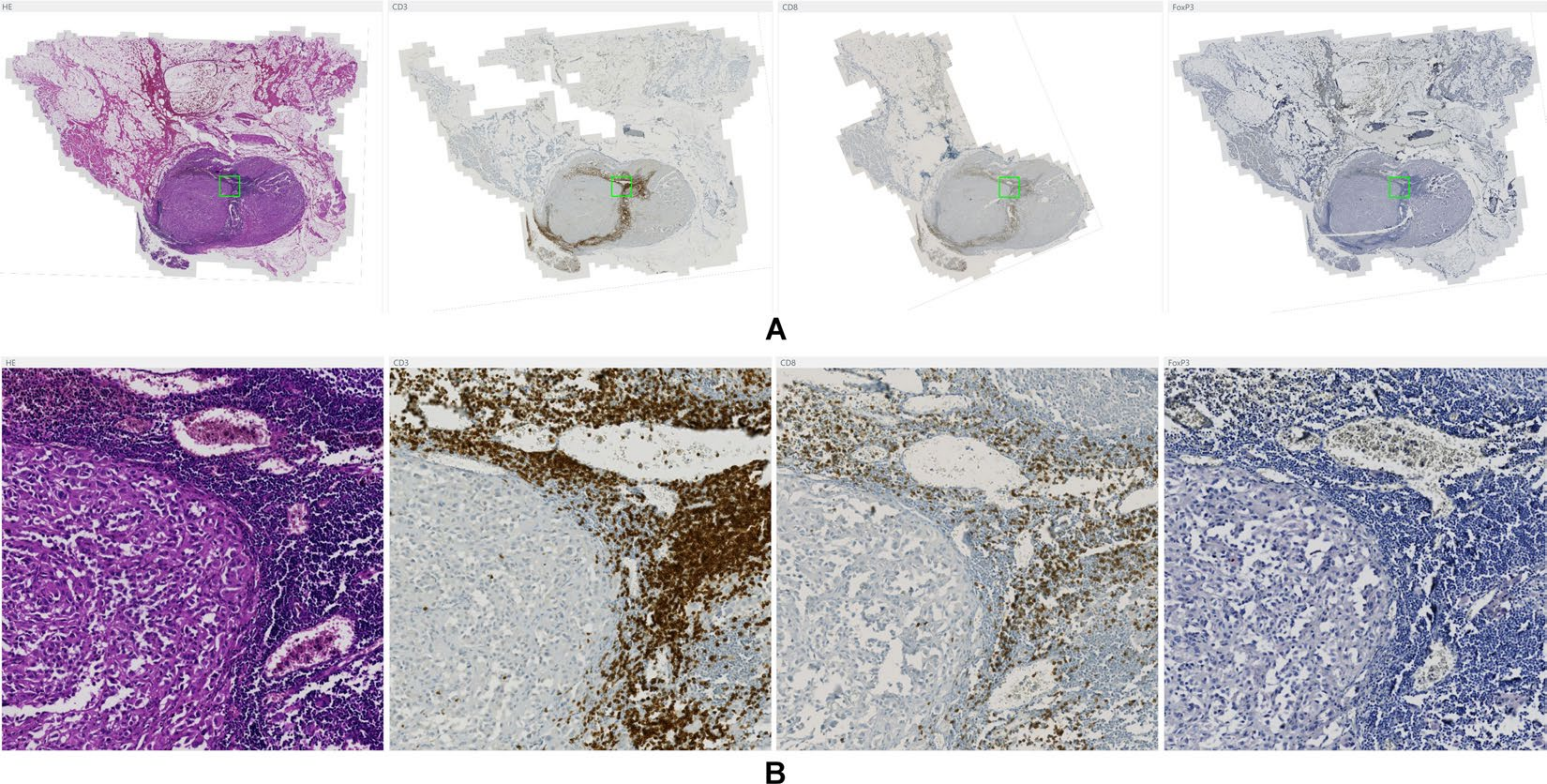
Example result of the co-registration approach for four stains. (**A**) Whole-slide images with different stains (left to right): H&E, CD3, CD8, FoxP3. The green box highlights the position of zoomed regions. (**B**) Zoomed regions from WSIs shown in panel (A) with stains in the same order as in (**A**).

To reconstruct the original region topology of consecutive physical tissue sections, the respective images needed to be transformed into a common coordinate system. To this end, we used a multi-modal co-registration approach^20^. This approach first normalizes the differently stained input images using minimum intensity projection along the RGB color channels followed by intensity inversion. After an initial coarse alignment of down-sampled versions of the images by rigid registration and correspondence finding in case of multiple tissue parts, a multi-resolution registration scheme is employed. The approach uses the normalized cross-correlation as similarity metric and subdivides local image patches depending on their content. Once the resolution specified by the user is reached, the method terminates and computes a regular grid of correspondence points (landmarks) for which the transformation parameters are obtained. As shown by the example result in Fig. 3 the method is capable of correctly aligning highly dissimilar tissue sections as caused by tissue rupturing or folding during the slide preparation process. For more details on the method see Yigitsoy *et al*.^20^.

### Deep learning-based region classification

A major difficulty in our data set was to robustly distinguish immunohistochemically stained CD3+ and CD8+ cells from melanin. Since brown stain was used for CD3 and CD8, and also melanin appears in different shades of brown, it is a non-trivial problem to robustly un-mix both phenotypes (see Supplemental Fig. S2). However, for accurate quantification of IHC-positive cell densities it was crucial to avoid false positives caused by melanin misclassified as IHC-positive cells. An explicit formulation of appropriate features separating these two classes is not straightforward, and consequently, we chose to use a convolutional neural network to address this task. Since annotating a sufficiently large amount of training data is a tedious and time-consuming process we developed a method to generate training data automatically from possibly clear cases. This dramatically reduced the amount of manual interaction and only a quick visual inspection and correction of the extracted training data was necessary.

#### Automatic training data generation

**Figure 4:**
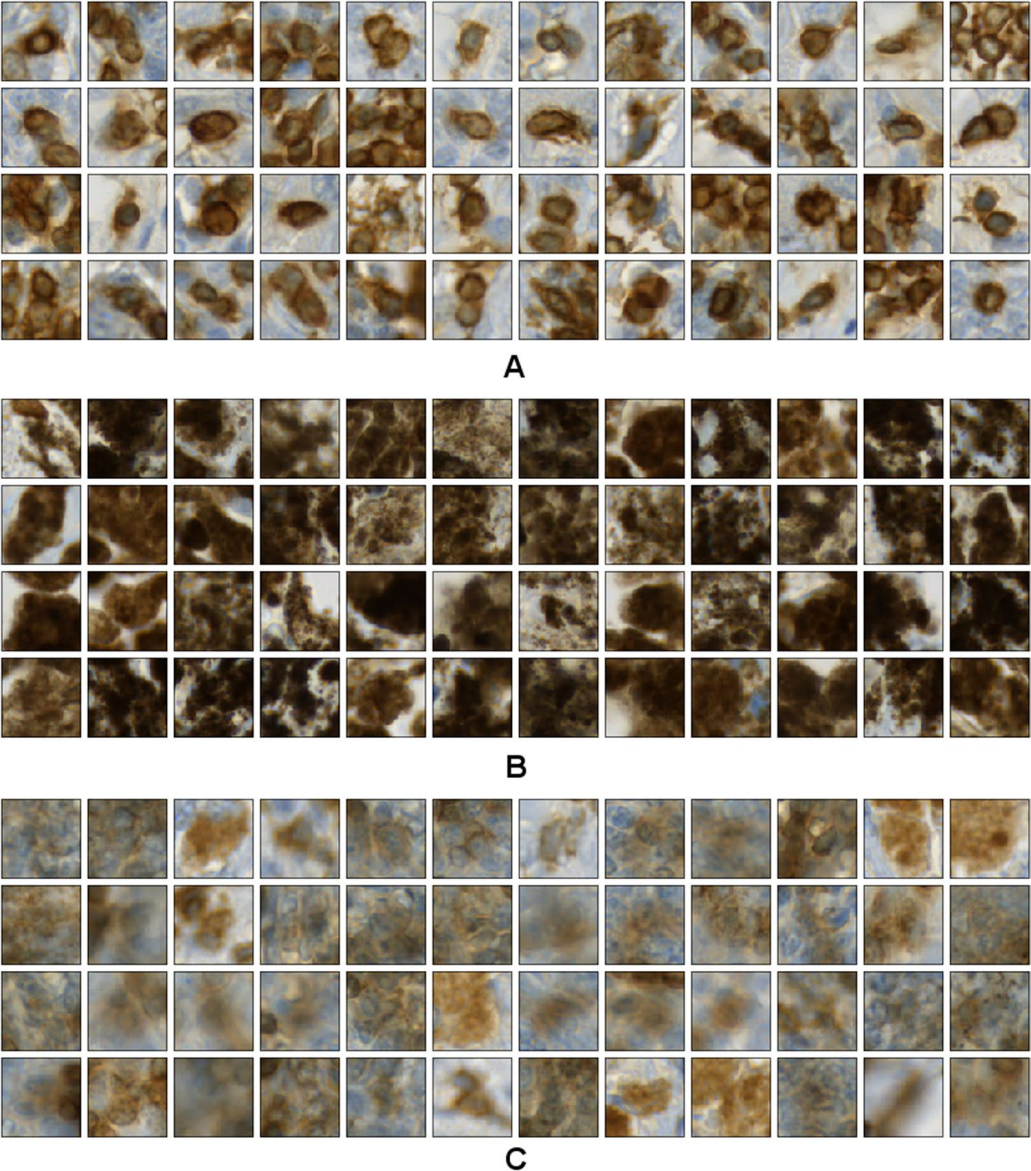
Training data example patches for the three considered classes: (**A**) CD3+ nuclei, (**B**) melanin, and (**C**) non-specific stain.

Convolutional neural networks (CNNs) gained enormous attention within the past couple of years as they started to help solving so far un-solved problems^21^ and tremendously pushed the limits of data analysis accuracies compared to previous methods^22^. However, as for all learning-based methods, the performance of CNNs highly depends on the amount and quality of the available training data. To provide a sufficiently large amount of high-quality labeled training data for the CD3+ and CD8+ versus melanin classification problem, we developed an automated method for training data generation (see Supplemental Code). This method combines the information from the two co-registered IHC channels CD3 and FoxP3, where training patches were extracted from the CD3 channel and FoxP3 was employed to detect melanin-positive regions. To this end, we used the cell nucleus segmentation result for brown-stained objects computed on the FoxP3 section and transformed it to the CD3 coordinate system using the registration parameters obtained by the co-registration procedure (see Supplemental Fig. S3B,E). Note that on FoxP3 sections brown-stained objects exclusively corresponded to melanin as FoxP3+ nuclei were stained in red. Training patches for the class *CD3*+ were extracted only from those regions which showed IHC-positive cells in the CD3 segmentation result but no brown-stained objects in the FoxP3 segmentation result to avoid using regions including melanin (see, e.g., Supplemental Fig. S3D,E, top-left). On the other hand, we extracted training patches for the class *melanin* in regions with brown-stained objects in the FoxP3 segmentation and without IHC-positive cells in the CD3 segmentation (see Supplemental Fig. S3D,E, bottom-right). As a third class, we defined *non-specific stain* including brown regions which did neither corresponded to correct IHC-positive cells in CD3 nor to melanin in FoxP3. Such stain was automatically identified by its faint appearance. We chose a patch size of 80 × 80 pixels^2^ (i.e. 17.6 × 17.6 µm^2^), which typically contained one to three IHC-positive cells. With this patch size we yielded a sufficiently high resolution for regions with intermixed IHC-positive cells and melanin in the prediction stage, while at the same time providing sufficient context for the CNN. Example patches for all classes are shown in Fig. 4.

In total 83997 training patches were extracted from a subset of 16 patients. Next, the data was visually inspected by browsing through gallery views of the patches and mislabeled patches were excluded from the training data set, resulting in 63842 approved patches. However, since the number of patches per class was highly unbalanced, as shown in Table 3 (left, (1)), we artificially increased the number of patches in the underrepresented classes *melanin* and *non-specific stain* by data augmentation. For patch augmentation we used rotation (angles 0°, 90°, 180°, 270°) and four intensity transformations (histogram scaling), resulting in 15 additional variations per patch (see Supplemental Fig. S4). To obtain a balanced training set, samples were randomly drawn from the set of augmented patches for each underrepresented class, until a balanced class distribution was reached (see Table 3, left, (2)).

**Table 3:** Number of training patches per class.

Class	Complete training data set		Split into subsets	
	(1) before balancing	(2) after balancing	(3) Train	(4) Test
CD3	40310	40310	30109	10201
Melanin	20787	40304	32706	7598
Non-specific stain	2745	40251	34395	5856
**Total**	**63842**	**120865**	**97210**	**23655**

(Left) Complete training data set: (1) before and (2) after balancing the data based on data augmentation. (Right) Balanced data set split on patient level into training and test subsets for performance evaluation of the classifier: (3) Training set based on 12 patients, (4) test set based on 4 patients.

To enable a proper evaluation of the classifier performance we split the training data into a training and a test subset at patient level, where 12 patients were used for training and 4 patients were used for testing (see Table 3, right).

#### Convolutional neural network

**Figure 5:**
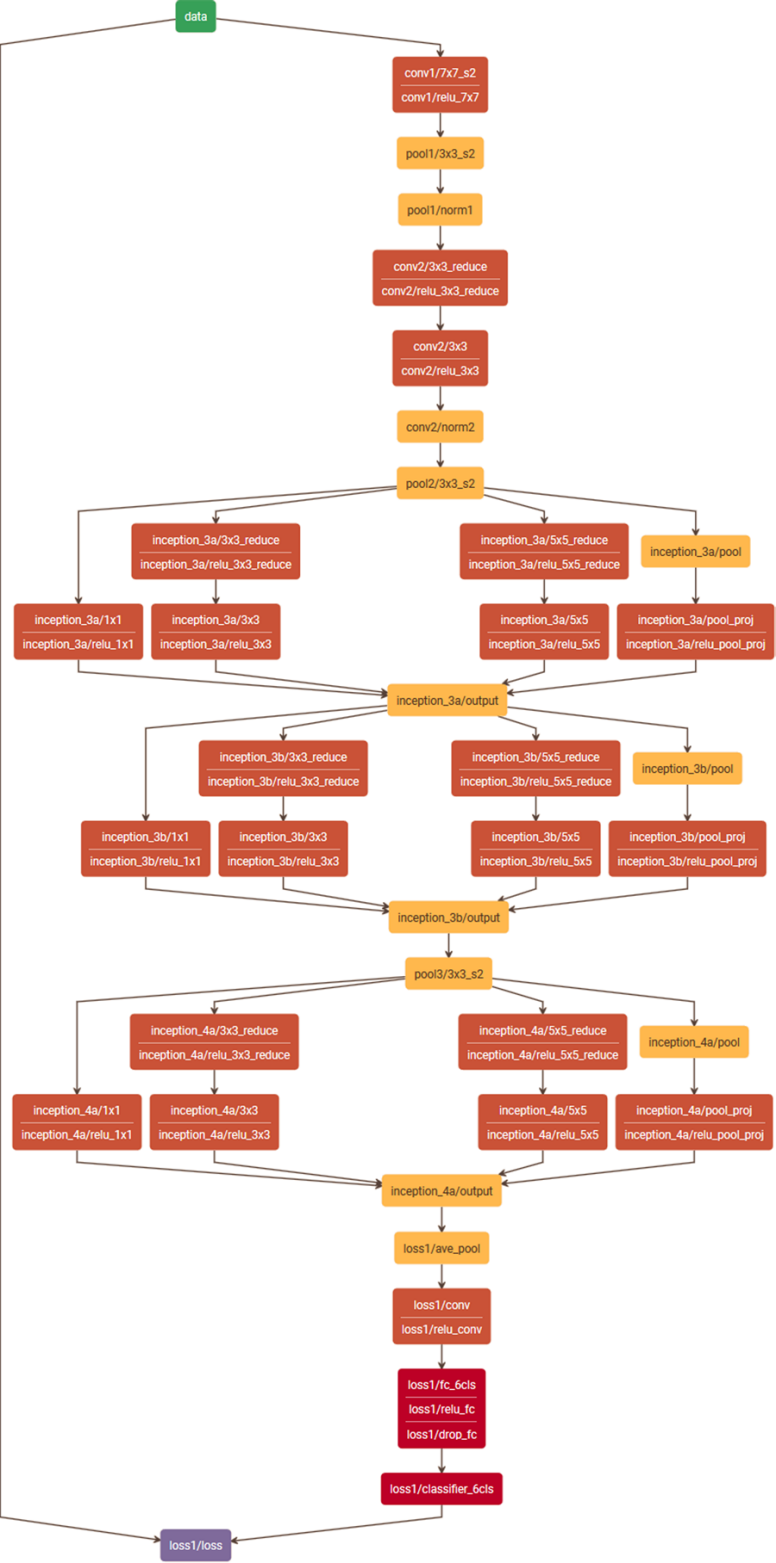
Reduced GoogleNet. The original network^23^ was cut at the first intermediate loss layer, resulting in a total of three inception units instead of nine as in the original network. (Plot generated with Netscope, http://ethereon.github.io/netscope/#/editor).

To classify regions into the classes *CD3*+, *melanin*, and *non-specific stain* we used a CNN based on the network architecture GoogleNet^23^. However, since the original GoogleNet has been developed for a much more complex task, i.e. the classification of natural images into 1000 distinct classes (ImageNet challenge ILSVRC14^22^), this network is characterized by a large number of around 6.7 M parameters to be optimized. For the three-class problem addressed here this huge network was unnecessary complex, and thus, we used a simplified version of this architecture. The original GoogleNet is built of nine inception modules in total, where after each three inception modules there is an auxiliary loss layer to include information from intermediate layers in the optimization process during training. We cut the network at the first intermediate loss layer, that is, after the first block of three inception modules (see Fig. 5) and used this loss layer as the new network output. Thus, we reduced the number of network parameters to about 2.5 M which was more appropriate given the complexity of our classification task and the number of available training patches. Moreover, we used precomputed weights from pretraining of the network with the ImageNet data^22^ for the convolutional layers. Only the fully connected layers at the output of the network were trained from scratch while the weights of all other layers were refined (transfer learning).

For performance evaluation of the network we trained on the training subset including 97.2 k patches and tested on the validation subset including 23.7 k patches (see Table 3, right). We ran the training for 250 k iterations using the stochastic gradient descent (SGD) solver for optimization. The training curve as well as the resulting accuracies are shown in section **Results** below. After performance evaluation, we trained the final network for application to the whole-slide images on all 120.9 k available training data patches using the same settings as before (250 k iterations, SGD solver).

**Figure 6:**
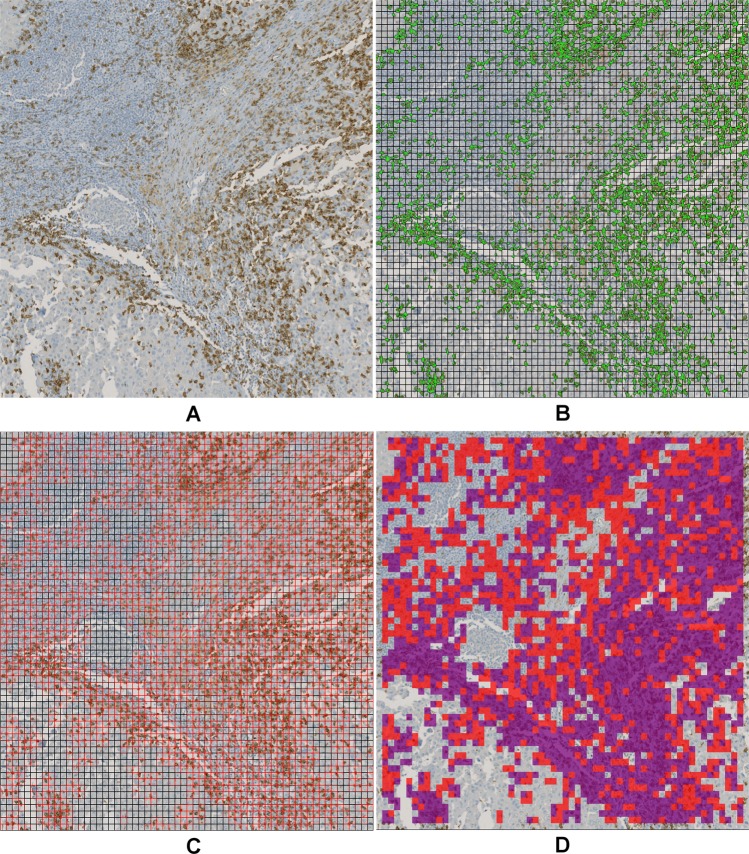
Application of trained CNN to whole-slide images in an optimized patch-based approach. (**A**) Raw image including CD3+ cells and non-specific stain, (**B**) overlay of the cell segmentation with potential CD3+ cells in green and 80 × 80 pixel grid, (**C**) relevant patches (red) identified based on positive cells in (**B**,**D**) prediction result of the CNN (violet: CD3+, red: non-specific stain).

Finally, we applied the final network to the whole-slide images (WSIs) stained for CD3 and CD8 for patch-wise prediction, corresponding to a stride of 80 pixels. To accelerate the prediction step we pre-selected the relevant candidate patches and performed prediction only on the selected patches. Relevant patches were identified based on the IHC-positive nucleus segmentation described above. First, WSIs were split into regions based on a regular grid and only regions inside the tissue were further processed. For each region, the IHC-positive cell segmentation was loaded and the region was split again into patches of size 80 × 80 pixels^2^. Patches overlapping with IHC-positive cell segmentations were processed by the CNN for prediction and the obtained class was stored in a prediction heatmap where each heatmap pixel represents one patch (see Fig. 6).

### Image feature extraction

As potential signatures for ipilimumab response prediction a set of image-based features was extracted based on all regions-of-interest (ROIs) and across consecutive tissue sections stained for CD3 and CD8. To this end, we first defined the ROIs based on manual annotations of the tumor region, and second, computed local image features.

#### Region-of-interest definition

**Figure 7:**
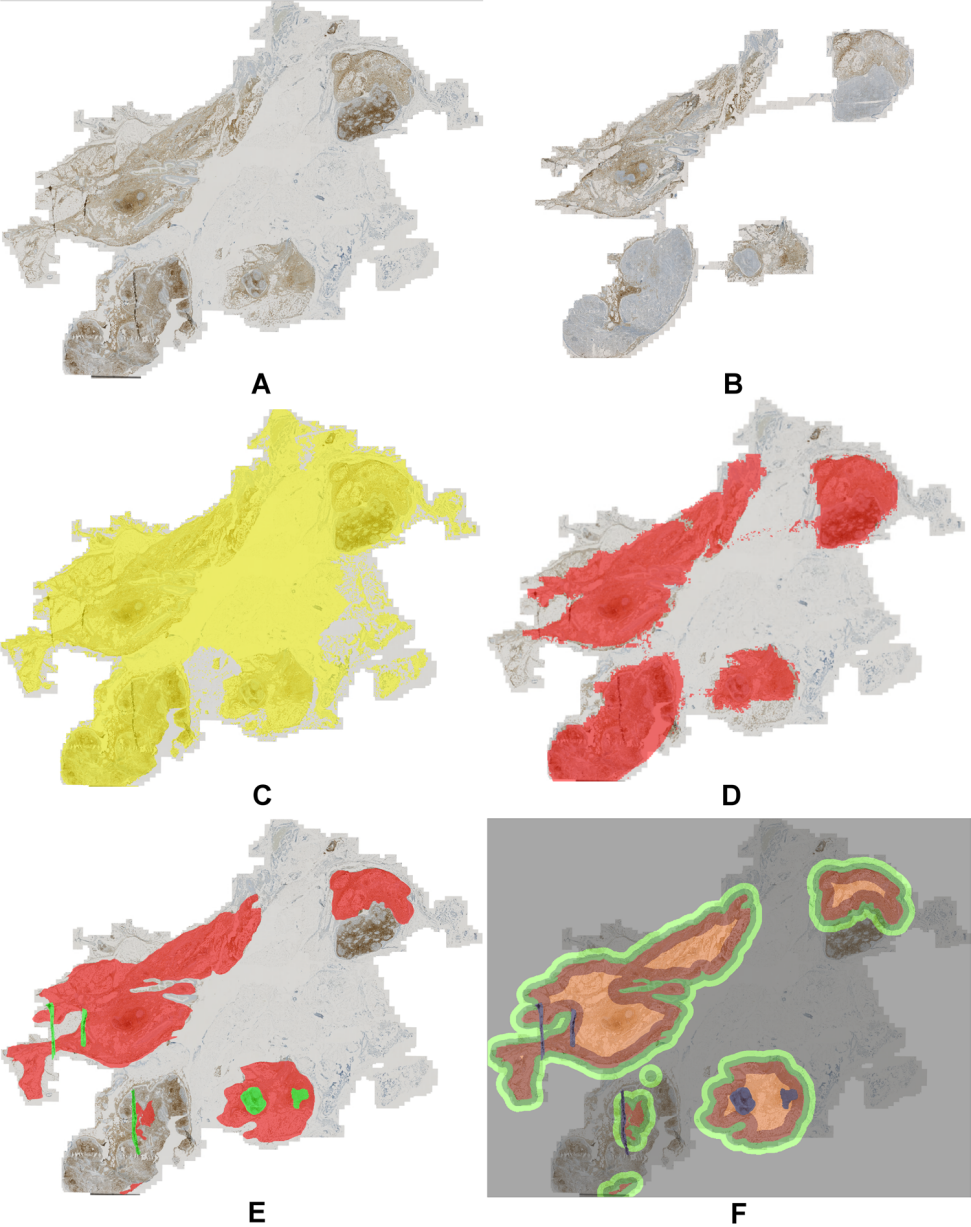
Region-of-interest definition for region-based analysis. (**A**) Raw image CD3, (**B**) raw image CD8, (**C**) tissue segmentation CD3, (**D**) tissue segmentation CD8, co-registered and overlaid to CD3, (**E**) manual annotation of tumor region (red) and exclusion areas (green), (**F**) regions-of-interest created by dilation: TME small (dark green) and large (light green), and created by erosion: ITI (*intra-tumoral infiltration region*, dark red), and tumor core (light red).

To characterize the immune contexture of a patient’s disease possibly precisely it is important to consider different compartments of the affected tissue separately. In particular, we considered the tumor core region, the tumor microenvironment, and the surrounding stroma as different functional entities regarding the tumor immune response. To avoid measurements outside of the actual tissue region we first performed segmentation of the tissue for both consecutive sections (CD3 and CD8) and discarded background regions from any further analysis. For sections with highly dissimilar tissue regions (see Fig. 7A–D) we only used the intersection of both co-registered tissue segmentations for extracting across-section features and each separate tissue segmentation for within-section features. As a basis for accurate definition of the ROIs we collected manual annotations of the tumor regions and exclusion areas such as necrosis or tissue artifacts (see Fig. 7E). The annotations were performed by an expert pathologist based on the CD3-stained digital images while also considering the H&E images. Using these annotations, we automatically computed different region types partitioning the tumor microenvironment (TME): the small TME (width 338 µm) and the large TME (width 676 µm) surrounding the tumor core were determined by morphological dilation of the annotated tumor region, and the intra-tumoral infiltration (ITI) region as a margin inside of the tumor (width 676 µm) was obtained by erosion of the annotated tumor region (see Fig. 7F). The choice of the TME sizes of 338 µm and its double 676 µm was inspired by the Immunoscore for colorectal cancer which uses an invasive margin spanning 360 µm into the tumor region as well as 360 µm into the surrounding tissue outside of the tumor^24^.

#### Region-based feature extraction

For region-based feature extraction we first quantified the number of pixels corresponding to IHC-positive nuclei in each ROI using the prediction heatmap obtained from the CNN to exclude melanin regions from the nucleus segmentation, and second, quantified the area of all ROIs in terms of heatmap pixels (i.e. 80 × 80 pixel^2^ patches). Note that the IHC-positive pixels were obtained using the cell nucleus segmentation approach (Brieu *et al*.^18^, section *Cell nucleus detection and segmentation*) which internally normalizes for staining variations between slides, and thus, resulted in highly robust readouts. In total, we considered six ROIs: *tumor*, *stroma*, *tumor plus stroma*, *TME small*, *TME large*, and *ITI (intra-tumoral infiltration region*, definition see above). Next, we computed the density of IHC-positive pixels per ROI by dividing the absolute number of IHC-positive pixels by the region size. The idea behind these local density measurements was to identify systematic patterns in the distribution of immune cells in different tissue compartments, particularly focusing on the TME. Based on the computed densities we also derived ratios of all combinations of densities to relate the immune cell distribution characteristics of individual ROIs within a patient’s WSI to each other. Finally, we computed average distances of IHC-positive cell nuclei of different IHC stains, i.e. the average distances of CD3+ to CD8+ and vice versa. This quantification of cell-to-cell distances enabled us to study potential systematic co-occurrence patterns which may provide insights in cell type-specific interactions. As the most relevant cell cluster size was not known, different neighborhoods were considered, in particular, we computed average distances to 2, 4, and 8 nearest neighbors. Overall, a moderately sized set of 114 features was extracted for each WSI.

### Feature and model selection with Monte Carlo cross validation

After feature extraction, we first performed a general basic data evaluation using median feature values as cut points for patient stratification into ipilimumab responders and non-responders. Next, we applied cut point optimization to derive more accurate uni-variate models for patient stratification and identified a relevant feature subset for robust stratification by comparing different feature selection methods in a cross validation framework. Finally, we applied and compared different classifiers which allowed us to combine multiple features for multi-variate stratification. Note that considering the relatively small set of 31 patients in this study, even the moderately sized set of 114 features is too large for deriving statistically meaningful readouts when optimizing over all features for patient stratification due to potential overfitting of the parameters to be optimized. For this reason we used a Monte Carlo cross validation approach to reliably estimate and systematically compare the stratification performance given different feature subset selection methods and different classification approaches.

#### Monte Carlo cross validation (MCCV)

**Figure 8:**
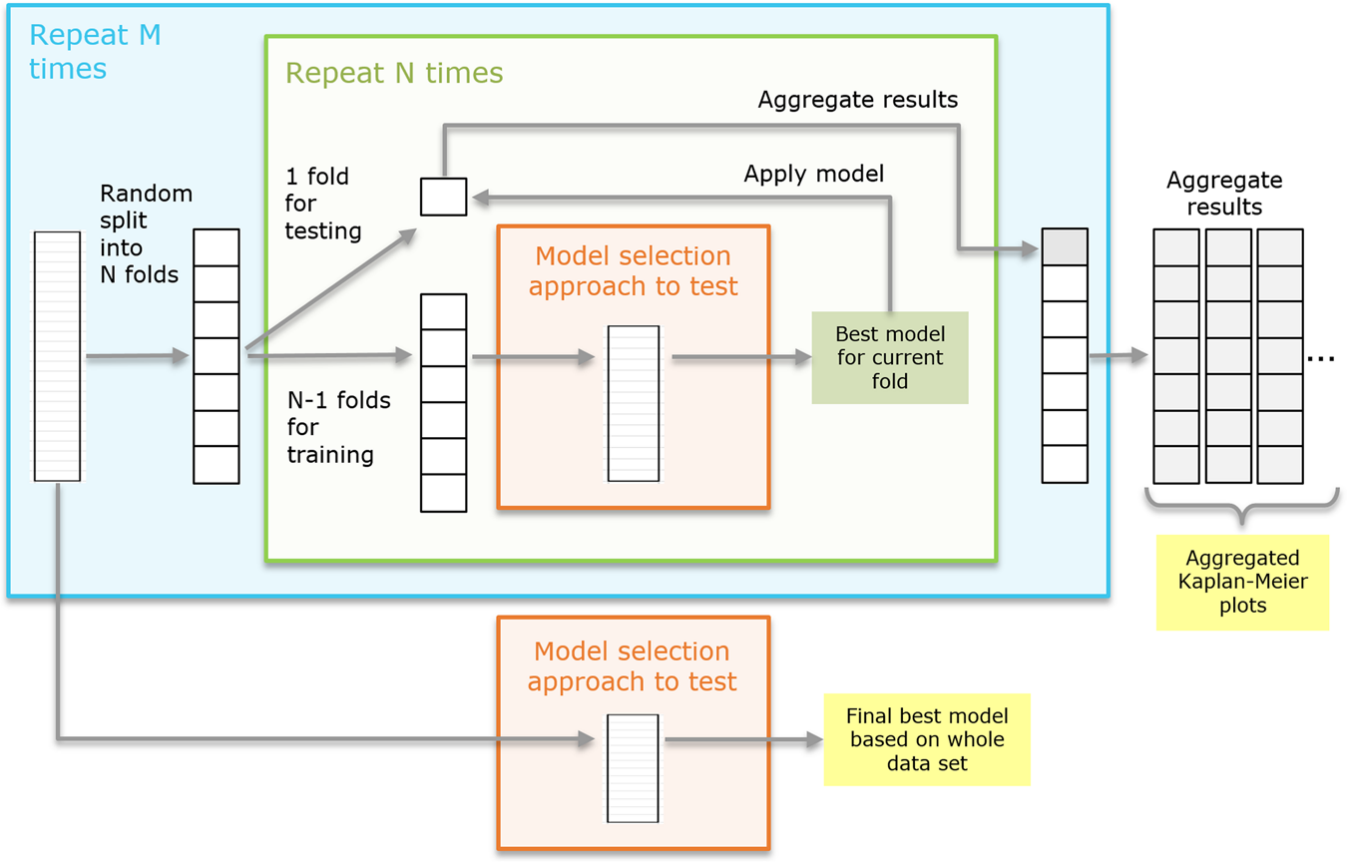
Monte Carlo cross validation workflow. Outer Monte Carlo cross validation loop (blue box) with nested inner N-fold cross validation (green box) to estimate the performance of a feature or model selection method (orange box). Finally, the model selection method is applied to the whole data set to obtain the final result.

Our Monte Carlo cross validation approach is characterized by three nested modules: (1) the outer Monte Carlo loop where the full data set is repeatedly subdivided randomly into N subsets, (2) the nested N-fold cross validation loop where in each run one fold is kept for testing while training is performed on the remaining N-1 folds, and (3) the inner-most module containing the actual feature selection or model optimization. A detailed sketch of the method is given in Fig. 8. The result of the inner-most module (model selection), being the best model for the given training data of N-1 subsets, is applied to the remaining test fold. The resulting prediction on the test fold is saved and collected over all N cross validation runs of the current Monte Carlo run. With M-fold repetition of the outer-most loop a set of aggregated predictions is created (see Fig. 8, right). Based on those aggregated predictions we compute aggregated Kaplan-Meier plots providing a visual impression of the robustness and performance of the tested model selection approach. In addition, the stratification performance for each aggregated prediction on the test folds is computed and used for comparison of different methods (see section **Results** below). In this work, we used *M* = 50 and *N* = 10. Finally, the best model selection approach is selected and applied to the complete data set to obtain the final model for application to new data (see Fig. 8, bottom).

#### Feature selection methods for uni-variate model selection

Using the described MCCV approach we performed a systematic evaluation of different feature selection methods to identify the most robust method given our data. In particular, we compared cut point optimization-based stratification using (1) the whole data set, (2) bootstrapping, (3) leave-one-out cross validation, (4) 10-times repeated 8-fold cross validation, (5) 10-times repeated 5-fold cross validation, (6) 10-times repeated 5-fold cross validation using the minimum p-value as objective function. For method (1) *stratification on the whole data set* we used all available patients and systematically tested all possible thresholds (cut points) for each feature regarding their stratification performance with a constraint on the minimum number of samples per class. Performance was measured in terms of the accuracy of predicting ipilimumab response for each threshold and feature. Finally, the best performing combination of feature and threshold was selected. In method (2) *bootstrapping*, instead of using all patients which is prone to overfitting, we repeatedly sampled random subsets of patients containing 95% of the data with multiple repetitions. The same cut point optimization as described for (1) was done on each bootstrap subset, and the feature which was among the *k* best features of a subset most often across all bootstrap runs was finally selected (we used *k* = 5). To compute the corresponding final threshold, all data of the current MCCV fold was used. In method (3) *leave-one-out cross validation* was performed where in each fold one patient was left out for testing while cut point optimization was performed on all remaining patients as described in (1). In each iteration, the response for the left-out sample was predicted for each feature with its respective best threshold. Thus, after processing all cross validation folds, a full prediction for all patients was available for each feature. The feature with the overall best prediction accuracy on the aggregated test folds was selected and, similarly as for method (2), the final threshold was computed based on all data of the current MCCV fold. The remaining feature selection methods (4–6) *M-times repeated N-fold cross validation* were all following the same concept as the surrounding MCCV as described above. A random split into *N* subsets was repeated *M*-times in an outer loop. In a nested inner loop *N*-fold cross validation was performed. Similar as for method (3) cut points were optimized on the training fold while response prediction was performed for the test fold with all features using their individual best thresholds. After aggregating the predictions over all cross validation folds the *k* best features were selected based on the aggregated predictions per *M*-run. Finally, the feature which occurred within the top *k*-ranked features most often over all *M* outer runs was selected as best model and the corresponding threshold was computed on all data of the current MCCV fold (we used *k* = 5). The difference between method (4) and (5) was the choice of different values *N* (8 versus 5), while for method (6) an alternative objective function for cut point optimization was used, i.e. minimizing the log-rank test p-value instead of maximizing the prediction accuracy. For the comparison of all methods see **Results, Comparison of feature selection methods**. Based on the results of the comparison of all feature selection methods we identified the most robust feature selection method for our data set and applied it to all data to obtain the most discriminative feature subset.

#### Selection of the best multi-variate model

The optimal feature subset identified as described above was used next to test the performance of different multi-variate models and identify the best-suited model. In particular, we studied (1) classification trees (CART)^25^ with different configurations, (2) support vector machines (SVM)^26^ with different settings, (3) perceptron^27^, (4) logistic regression^28^ and (5) bi-variate cut point optimization with cross validation and different functions for combining both features. For (1) we tested different depths *d* ϵ {1,2,3} and varied the settings for the minimum number of samples at a split *s*_*min*_ ϵ {5, 8} and at the leaf nodes *l*_*min*_ ϵ {3, 5, 6}. Also, we compared two different information gain measures, i.e. *gini impurity* (Eq. (1)) and *cross entropy* (Eq. (2)) which are used in the tree construction process to select the feature and threshold yielding the largest information gain. The measures are defined as follows:

1 $$gini=1-\sum _{k}{p}_{mk}^{2}$$

2 $$cross\,entropy=-\sum _{k}{p}_{mk}{\rm{l}}{\rm{o}}{\rm{g}}({p}_{mk})$$

where *p*_*mk*_ is the proportion of class *k* observed in node *m*.

For (2) we used differently sized feature sets with 3, 5, and 7 features, and a soft-margin SVM with linear kernel. For methods (3) and (4) a feature set including 5 features was used. In all cases the feature sets were subsets of the previously selected feature set where the subsets were picked in the order of their rank with respect to the average stratification accuracy.

In case of method (5) the *bi-variate cut point optimization*, we employed a similar framework as described above for uni-variate feature selection, i.e. we performed 10-times repeated 8-fold cross validation. However, in contrast to the above described method the decision here was based on two features where the corresponding predictions were combined using logical operations (AND, OR, XOR). The cut points for both features were optimized given the combination function. We tested the performance of the bi-variate method using only the AND operation versus an optimization over all logical operations. The results of the comparison of multi-variate models are given in section **Results**. Finally, the best-performing model was trained on the whole data set to obtain the final best model.

### Software

The basic image analysis methods (sections **Basic Image Analysis** and *Automatic training data generation*) were implemented in Definiens Developer XD 2.5.0 (Definiens AG, Munich, Germany). For pseudo code descriptions see Harder *et al*.^15^ and Supplemental Code. All CNN-related methods (section *Convolutional neural network*) were developed using the C++ -based software framework Caffe^29^. The model definition file (prototxt) as well as the weight file (caffemodel) are available as supplemental material. The feature extraction (section **Image Feature extraction**, for pseudo code see Harder *et al*.^15^) and the Monte Carlo cross validation (section **Feature and model selection with Monte Carlo cross validation**) were implemented in a Definiens internal data mining platform using the Python package scikit-learn^30^ for the multi-variate models (1) *classification trees* (class sklearn.tree.DecisionTreeClassifier) and (2–4) *SVM*, *perceptron*, and *logistic regression* (class sklearn.linear_model.SGDClassifier).

### Ethical approval

The study was approved by the internal ethics board of the National Cancer Institute “G. Pascale” and all patients provided written informed consent at time of biopsy. All methods were performed in accordance with the relevant guidelines and regulations.

## Results

### CNN prediction accuracies

**Figure 9:**
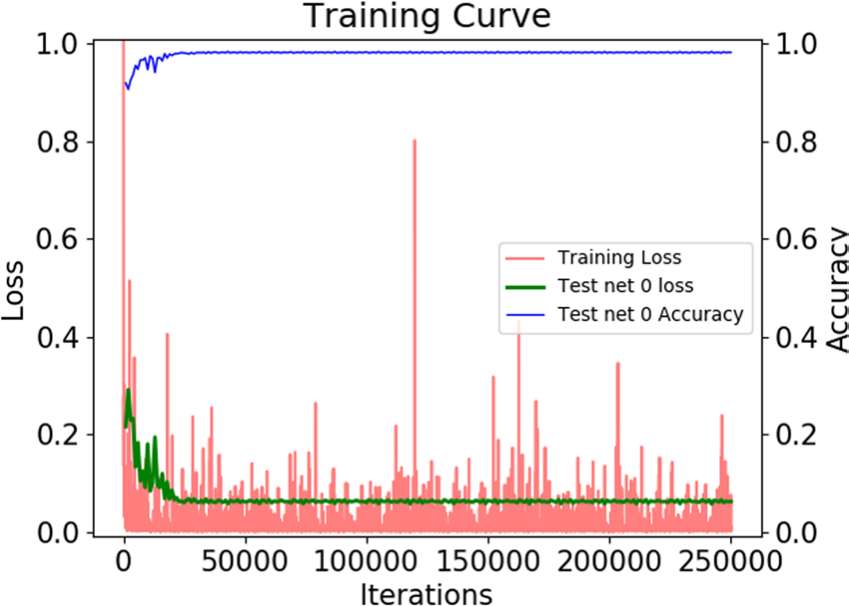
Training curve of the reduced GoogleNet trained with disjoint training and test subsets for performance evaluation. (Red) loss on the training set, (green) loss on the test set, (blue) accuracy on the test set.

To systematically quantify the performance of the reduced GoogleNet (see Fig. 5) we split our data into a disjoint training and test set at patient level (see section *Automatic training data generation*, Table 3). We trained the network for 250 k iterations on the training set using SGD and obtained the training curve shown in Fig. 9. We used a base learning rate of 0.001 with a step-wise decrease (stepsize: 10000), a weight decay of 0.002, and batch size of 50 samples. As can be seen in the training curve the loss and the accuracy on the training set converged after about 25 k iterations to a loss around 0.06 and an overall accuracy on the test set of about 98%. Performing a patch-wise prediction on the test set and evaluating the prediction with respect to the true class allowed us to set up the confusion matrix and compute the overall and per-class prediction accuracies (see Table 4). We obtained a high overall accuracy on the test set of 98.2% and per-class accuracies of 99.4%, 96.0%, and 99.0% for the classes *CD3*+, *melanin*, and *non-specific stain*, respectively.

**Table 4:** Confusion matrix for the prediction on the test set for performance evaluation.

True Class	Predicted Class			Accuracy per class
	CD3	Melanin	Non-specific	
CD3	10140	31	30	99.4%
Melanin	304	7291	3	96.0%
Non-specific	58	2	5796	99.0%

Rows provide the true class while columns show the class predicted by the network. The diagonal represents the correct classifications. The right-most column provides the accuracies per class.

### Comparison of feature selection methods

**Figure 10:**
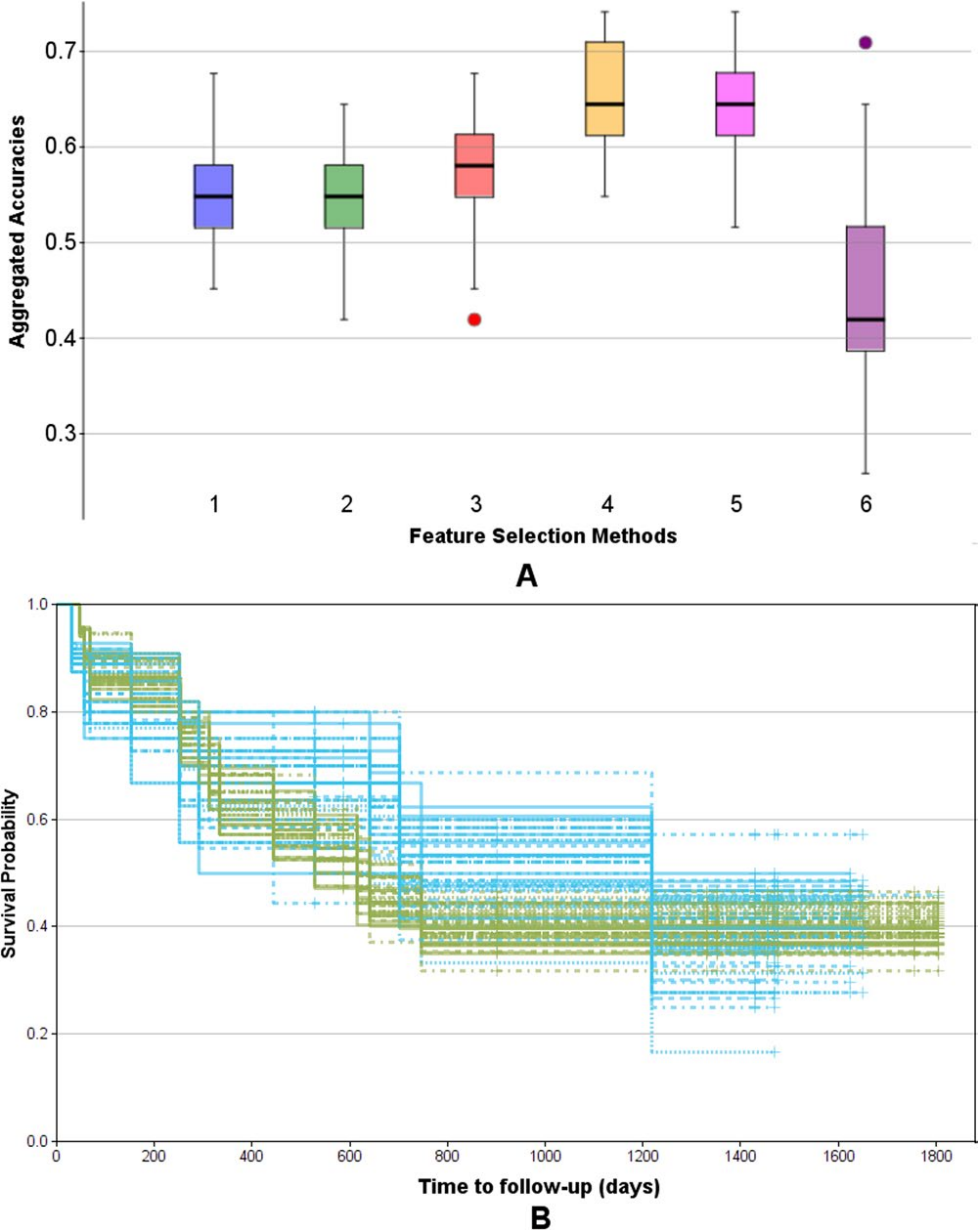
Evaluation of feature selection methods using the Monte Carlo cross validation (M = 50) for IPI response prediction. (**A**) Comparison of methods regarding their aggregated uni-variate stratification performance: (1) Stratification on whole data set (blue), (2) bootstrapping (green), (3) leave-one-out cross validation (red), (4) 10-times repeated 8-fold cross validation (yellow), (5) 10-times repeated 5-fold cross validation (magenta), (6) 10-times repeated 5-fold cross validation using the log-rank test p-value as objective function (violet). (**B**) Aggregated Kaplan-Meier plot (with respect to OS and stratification according to predicted IPI response) for the feature selection method (4) 10-times repeated 8-fold cross validation, yielding the highest accuracy. Blue: predicted response, green: predicted non-response.

The results of the comparison of different feature selection methods based on the Monte Carlo cross validation scheme are shown in Fig. 10A. Each box provides the distribution of accuracies on the test sets across all *M* = 50 Monte Carlo runs. To test the significance of our results we performed t-tests and non-parametric Mann-Whitney tests for all combinations of methods (see Supplemental Table S1). As can be seen in Fig. 10A the methods 10-times 8-fold cross validation (4, yellow) and 10-times 5-fold cross validation (5, magenta) clearly outperform the other methods (p_t-test_(3–4) = 5.43e-13, p_t-test_(3–5) = 9.68e-09), where (4) seems to be slightly but not significantly superior to (5) (p_t-test_(4–5) = 0.09). Interestingly, using all data (1, blue) performs similar as the bootstrapping approach (2, green) (p_t-test_(1–2) = 0.95), while the leave-one-out cross validation (3, red) yields significantly better results (p_t-test_(2–3) = 0.002). The 10-times 5-fold cross validation using the log-rank test p-value for cut point optimization is inferior to all other methods. This can be explained by the small cohort size, in particular, the small number of cases inside the cross validation folds. We also performed ANOVA over all methods, resulting in a highly significant p-value of 2.72e-51. Consequently, we selected approach (4) 10-times 8-fold cross validation with an average cross-validated stratification accuracy of 66.3% to perform our final feature selection on the whole data set. As a result we obtained the subset of 7 features and the respective cut points shown in Table 5 as uni-variate stratification models. For example, the top-performing model classifies those patients as ipilimumab *responders* having a feature value above 5.2 for the feature *ratio of CD8*+ *density in the ITI (intra-tumoral infiltration region) and CD8*+ *density in the stroma* (median OS 17.3 months vs. not reached). Furthermore, Table 5 provides the mean accuracies of the models on the test folds averaged over all 10 repetitions of the 8-fold cross validation as well as the frequency each model was among the best 5 models measured over all repetitions when applied on the whole data set. It can be seen that using the whole data set much higher cross-validated accuracies of up to 77.4% are yielded. This set of features was further used for multi-variate stratification as presented below. The aggregated Kaplan-Meier curves over all *M* = 50 runs of the MCCV for the selected best method are shown in Fig. 10B. Generally, the curves separate well, however, for the early time points as well as for the very late time points there is a certain amount of overlap, suggesting that the data might not be very well separable based on a simple uni-variate model.

**Table 5:** Feature set selected by 10 times 8-fold cross validation.

Model predicting response			Performance		Median OS [months]	
Feature	Dir	Thresh	Acc.	Count	<= Thresh	>Thresh
(1) Density CD8 *ITI */ Density CD8 *Stroma*	>	5.200	0.774	10	17.3	n.r.
(2) Density CD3 *Stroma */ Density CD8 *ITI*	<	0.615	0.742	10	40.0	20.2
(3) Density CD3 *TME large */ Density CD8 *TME small*	<	1.798	0.736	10	n.r.	14.6
(4) Density CD3 *Stroma */ Density CD8 *Tumor*	<	0.524	0.742	9	40.0	17.3
(5) Density CD8 *Tumor */ Density CD8 *TumorStroma*	>	1.349	0.742	7	14.6	n.r.
(6) Density CD3 *ITI */ Density CD3 *TumorStroma*	>	1.508	0.710	3	17.3	n.r.
(7) Density CD3 *TME large */ Density CD3 *TME small*	<	0.780	0.710	1	n.r.	17.3

(Left) The model for predicting *response* is defined by the combination of feature, direction (column Dir), and cut point (column Thresh). (Middle) Estimated performance of the model where Acc are the average accuracies on the test folds (averaged over 10 Monte Carlo runs of 8-fold cross validation) and Count provides the frequency a feature was among the top 5 over all 10 Monte Carlo runs. (Right) Median overall survival for stratification using Thresh (n.r.: not reached).

### Comparison of multi-variate models

**Figure 11:**
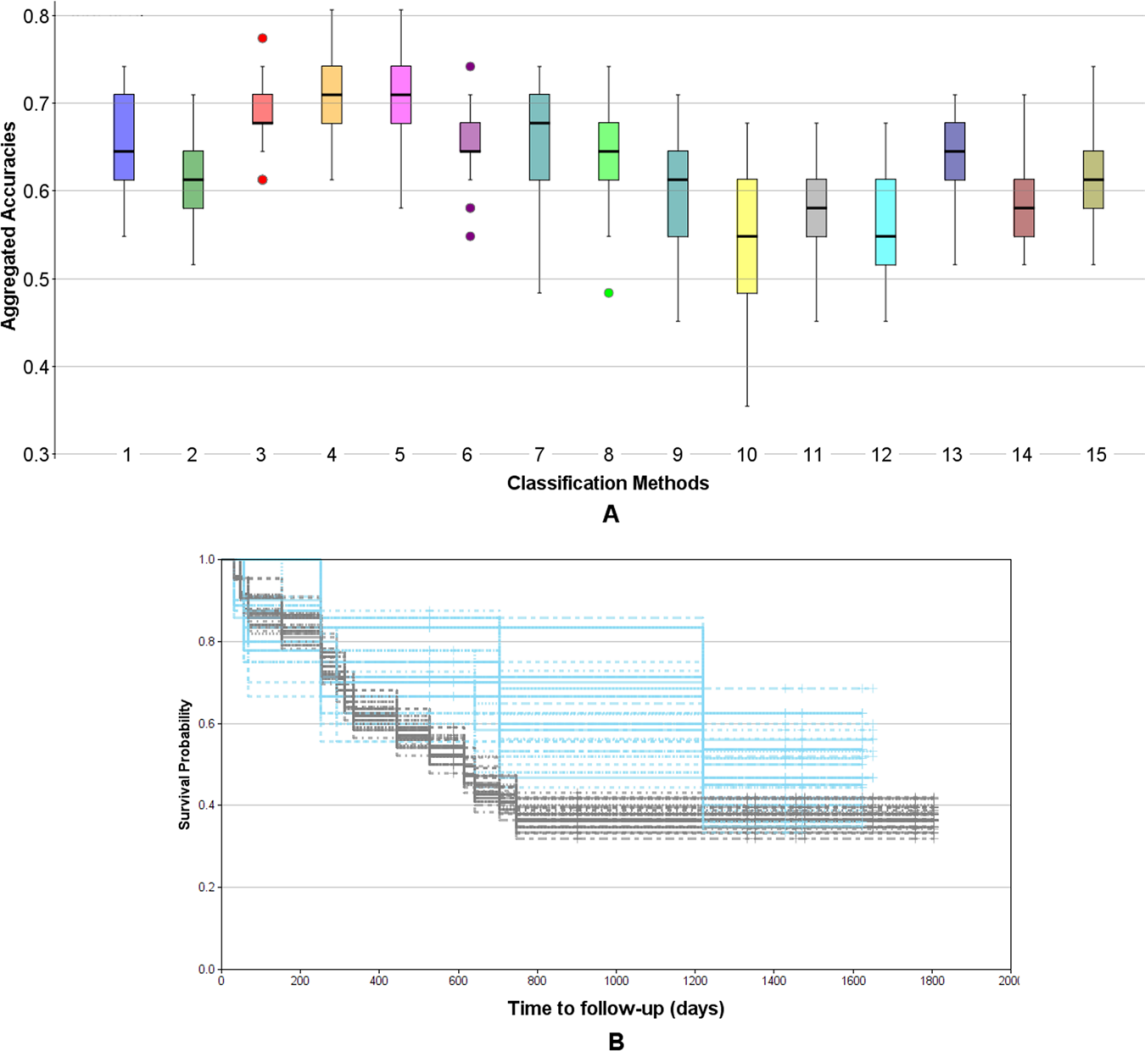
Evaluation of multi-variate models using the Monte Carlo cross validation (M = 50) for IPI response prediction. (**A**) Comparison of methods regarding their aggregated stratification performance: (1) uni-variate stratification with 10-times repeated 8-fold cross validation as reference, (2) tree (*d* = 1, *s*_*min*_ = 5, *l*_*min*_ = 3), (3) tree (*d* = 2, *s*_*min*_ = 5, *l*_*min*_ = 3), (4) tree (*d* = 2, *s*_*min*_ = 5, *l*_*min*_ = 5), (5) tree (*d* = 2, *s*_*min*_ = 5, *l*_*min*_ = 5), information gain measure: *cross entropy*, (6) tree (*d* = 2, *s*_*min*_ = 8, *l*_*min*_ = 6), (7) tree (*d* = 3, *s*_*min*_ = 5, *l*_*min*_ = 3), (8) SVM, 5 features, hinge, (9) SVM, 3 features, hinge, (10) SVM, 5 features, hinge sqr, (11) SVM, 7 features, hinge, (12) perceptron, 5 features, (13) logistic regression, 5 feat, (14) bi-variate stratifier with 10-times repeated 8-fold cross validation (all functions), (15) bi-variate stratifier with 10-times repeated 8-fold cross validation (only AND). (**B**) Aggregated Kaplan-Meier plot (with respect to OS and stratification according to predicted IPI response) for the method (4) tree (*d* = 2, *s*_*min*_ = 5, *l*_*min*_ = 5) yielding the highest accuracy. Blue: predicted response, gray: predicted non-response.

**Figure 12:**
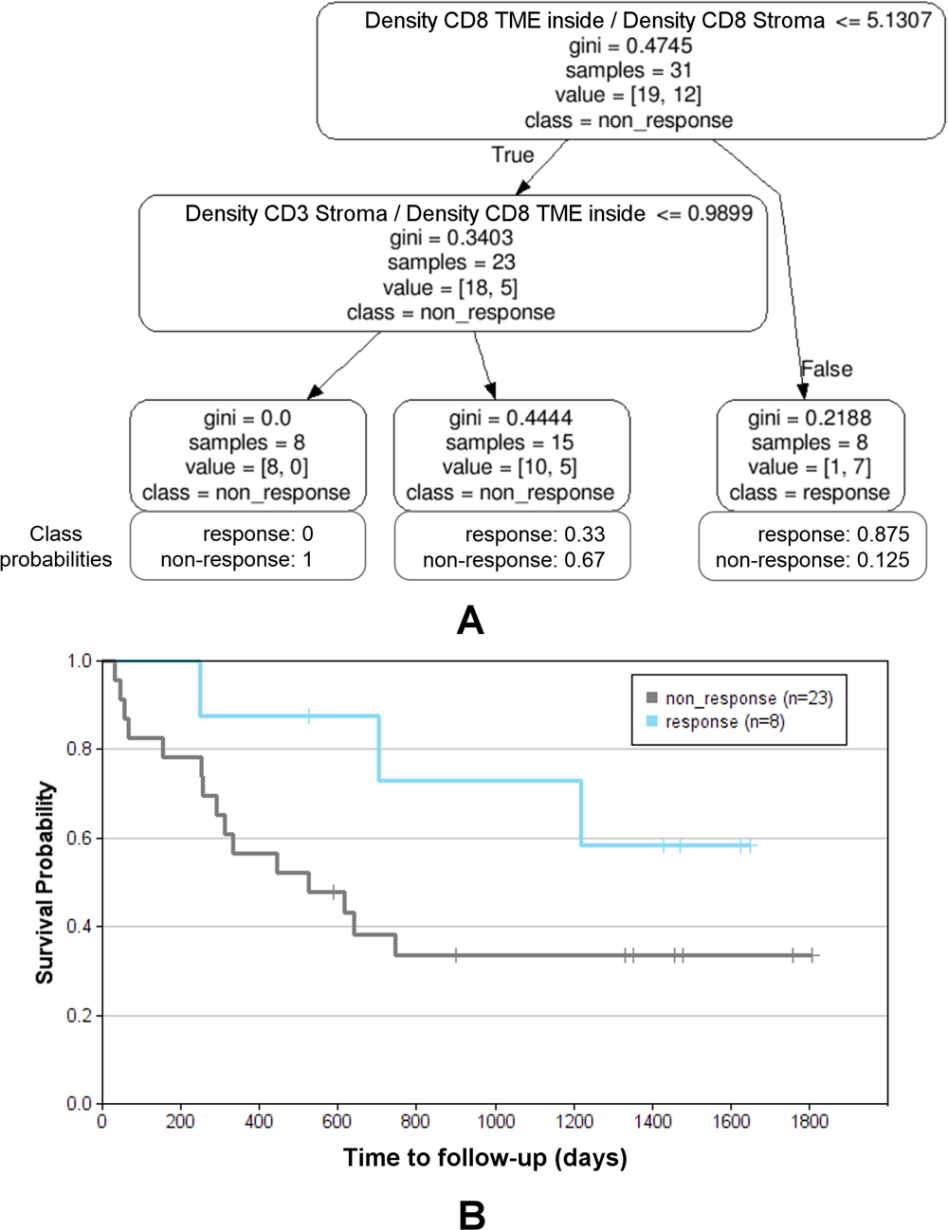
Final decision tree for IPI response prediction trained on all data. (**A**) Decision tree model (*d* = 2, *s*_*min*_ = 5, *l*_*min*_ = 5, information gain measure: *gini impurity*) with class probabilities providing the confidence at each leaf node. (**B**) Kaplan-Meier plot (with respect to OS and stratification according to predicted IPI response) for the application of the final tree model to whole data set (accuracy 80.7%). Blue: predicted response, gray: predicted non-response.

The results of the comparison of different multi-variate models using the Monte Carlo cross validation scheme are presented in Fig. 11A. The box plots provide the distributions of accuracies on the test sets over all *M* = 50 Monte Carlo runs. T-tests and Mann-Whitney tests were performed for all combinations of methods (see Supplemental Table S2) as well as ANOVA including all models (p_ANOVA_ = 1.71e-91). It turned out that methods (4) and (5), corresponding to decision trees of depth *d* = 2 with *s*_*min*_ = 5 and *l*_*min*_ = 5 showed the highest robustness and therefore yielded the best accuracies of all methods. As shown in Supplemental Table S2 the accuracies for method (4) differed significantly from any other method (p_t-test_(4-others) in [0.006, 5.38e-23] for all pairwise comparisons) except for method (5) (p_t-test_(4–5) = 0.298). This is not surprising as the only difference between (4) and (5) was the choice of the information gain measure, being *gini impurity* for (4) and *cross entropy* for (5). Method (3) was only slightly worse, differing from (4) only by a lower minimum sample number at the leaf nodes *l*_*min*_ = 3 with p_t-test_(3–4) = 0.006 and p_t-test_(3–5) = 0.087. Higher values for *l*_*min*_ and *s*_*min*_ seemed to be too restrictive as shown by the lower performance of model (6), similarly as using a tree of insufficient depth (see model (2)). Allowing a larger depth, on the other hand, obviously offered too much degree of freedom leading to overfitting and thus, less robust results in the MCCV framework (see model (7)). Among the other multi-variate models the best performance was yielded by methods (8) SVM with 5 features, and (13) logistic regression with 5 features. However, only for the logistic regression the achieved accuracies differed significantly from using the simple uni-variate model shown in (1) (p_t-test_(1–8) = 0.12 and p_t-test_(1–13) = 0.02). Based on our findings we selected method (4) as the most robust, and thus, best performing approach as the final model with an average Monte Carlo cross-validated accuracy of 70.9%. Figure 11B shows the aggregated Kaplan-Meier plot over all *M* = 50 runs of the MCCV. Compared to the Kaplan-Meier plot for the uni-variate model shown in Fig. 10B it is clearly visible that the multi-variate model provides a better stratification. Finally, we trained a decision tree model with the respective settings (*d* = 2, *s*_*min*_ = 5, *l*_*min*_ = 5, information gain: *gini*) on the whole data set as our final best model. The resulting tree model is shown in Fig. 12A. For each node, the uni-variate model for the node decision is given, as well as the value of the information gain, the total number of training samples at the node, the true class distribution, and the output class of the node. As can be seen, two ratio features were selected by the tree, i.e. the *ratio of CD8*+ *density in the ITI (intra-tumoral infiltration region) and CD8*+ *density in the stroma* (median OS 17.3 months vs. not reached) (being also the best-performing uni-variate model, see Table 5) and the *ratio of CD3*+ *density in the stroma and CD8*+ *density in the ITI (intra-tumoral infiltration region)* (median OS 40.0 vs. 20.2 months). At the three leaf nodes we observe three different scenarios: (1) clear classification as *non-responder* with a confidence of 100% (left-most node), (2) relatively clear classification as *responder* with a confidence of 87.5% (right-most node), and (3) unsure classification with slightly higher confidence for class *non-response* of 67% (middle node). However, note that the a-priori probability for our data set is 38.7% for *response* and 61.3% for *non-response*, and thus, the confidence for *non-response* at the middle node almost equals the respective a-priori probability. This tree architecture has the advantage that unclear samples are classified in the unsure class rather than being assigned to one of the other classes without confidence, and thus, offers the option to further analyze such unclear samples. Figure 12B shows the Kaplan-Meier plot of the predicted stratification when applying the final decision tree model shown in Fig. 12A to the complete data set (median OS 17.3 months vs. not reached). It can be seen that both arms are well separated and that the curve resembles the true stratification in *responders* versus *non-responders* shown in Supplemental Fig. S1A relatively closely with an overall accuracy of 80.7%. However, this basically shows how well the model manages to represent the training data and does not necessarily provide a reliable estimate for the expected performance on new data. For this we refer to the aggregated Kaplan-Meier plot in Fig. 11B.

### General data analysis using median cut points

Besides the robustness analysis with optimal feature subset selection and model selection we also performed a standard median cut point analysis. This analysis naturally does not yield very high stratification accuracies for our data since the cohort is not balanced regarding responders (12) and non-responders (19). However, it allowed us to compare general trends in our relatively small data set with findings obtained in the related MISIPI study^14^ for basic validation of our data. Note that for MISIPI the studied patient cohort was much larger (with IHC measurements for 77 patients) and a broader range of immunohistochemical markers was used (CD3, CD8, CD163, PD-L1, FoxP3). Since in our study, e.g., PD-L1 was not available the results could not be directly compared. Nevertheless, we observed similar trends in our data regarding the markers used in both studies. In particular, similarly as observed in Madonna *et al*.^14^ our data indicates that patients with CD8+ density in the TME below the median had a longer median overall survival than patients with higher CD8+ density (median OS 23.1 vs. 14.6 months; p > 0.9). This non-significant trend is observed with both, the small (338 µm) and the large (676 µm) TME radius but is not visible when considering the whole tissue (median OS 23.1 vs. 24.5 months; p > 0.7) (see Supplemental Fig. S5A, rows 1 to 3). Using optimized cut points (see section *Feature selection methods for uni-variate model selection*, method (1)) instead of median cut points, the trend is even more prominent, in particular, for the whole tissue region and the large TME (Supplemental Fig. S5B). For the most robust discriminative feature discovered in this work, the median cut point analysis showed the same trend as discovered by the optimized cut points: patients with a high *ratio of CD8*+ *density in the ITI (intra-tumoral infiltration region) and CD8*+ *density in the stroma* had a longer median overall survival than patients with a low ratio (median OS 20.2 vs. 40.0 months; p > 0.5) (Supplemental Fig. S5, row 4). This only applies for the ratio, the nominator alone does not show a clear trend (Supplemental Fig. S5, row 5).

### Multi-variate models for survival prediction

To demonstrate the broad applicability of our approach we additionally investigated its performance for predicting the duration of survival according to the categories *short-term* for survival times of up to 12 months (n = 11) and *long-term* for survival times of above 12 months (n = 20). Here, we used the best feature selection method as determined in section **Comparison of feature selection methods** (i.e. 10-times 8-fold cross validation) and compared the best-performing multi-variate models identified in section **Comparison of multi-variate models** (i.e. decision tree methods (3), (4), (5), and (7), SVMs (8) and logistic regression (13)). We found that the decision tree methods (3), (4), and (7) performed best (see Supplemental Fig. S6 and Table S3) with average cross-validated accuracies of 63.4% to 66.2%, and overall accuracies of 87.1% to 90.3% on the whole data set.

## Discussion

The breakthrough for immunotherapy in oncology started with the approval of the anti-CTLA-4 monoclonal antibody ipilimumab for second-line treatment of advanced melanoma in 2011, and the following extension to first-line therapy in 2013. Next, the two PD-1 blockers nivolumab and pembrolizumab were approved as well for the treatment of non-resectable and metastatic malignant melanoma in 2015. In more recent studies^1^ it was shown that the combination therapy of PD-1 inhibitors and anti-CTLA-4 antibodies is even more successful for a certain group of patients and the combination therapy has been approved in 2016. However, there are still many patients showing innate resistance or acquire resistance to any approved immunotherapy, and thus, biomarkers predicting response to systemic therapy are required to optimize patient selection and sequencing of therapies^31^. In particular, for combination therapies predictive biomarkers and companion diagnostic tests are essential since occurring adverse events are typically more frequent and more severe as compared to monotherapies. Also, given the large number of possible combinations, predictive markers could help to handle the adequate stratification of patients to the most suited combination therapy. However, even for monotherapies to date there are no approved predictive biomarkers available for clinical use, although the topic is target of active research. Results from previous studies suggest that an immunologically more active tumor microenvironment (TME) is an important factor for therapy response^4^. This comes along with the findings that a high baseline expression of immune-related genes in the TME as well as increased mutational and neoantigen burden of a tumor are favorable for clinical response to immune checkpoint blockade^8,9^.

In this context the idea of transferring the Immunoscore^10^ developed for stage III colorectal cancer to malignant melanoma as a potential predictive marker was fostered^12,13^. The MISIPI study (“Melanoma ImmunoScore evaluation in patients treated with IPIlimumab”) represents the first attempt to test the predictive potential of the Immunoscore in malignant melanoma. We here analyzed a smaller retrospective study designed as a proof-of-concept in conjunction with the MISIPI study including 31 patients with digital images of different tissue types (lymph node, skin, sub-skin, muscle). Similar as used by the original Immunoscore in colorectal cancer we focused on CD3+ and CD8+ lymphocytes, yet based on a finer partitioning into different compartments of the TME. Note that the diversity of our patient cohort regarding tissue types is much higher than in the colorectal cancer case. By statistically sound systematic comparison of the predictive power of different features characterizing the immune contexture we identified the most robust and best performing signatures. The top-performing uni-variate models indicate that a high ratio of CD8+ density in the intra-tumoral infiltration region to CD8+ or CD3+ densities in the surrounding tissue is associated to longer median OS and potential clinical therapy response (Table 5, features (1), (2)). This suggests that high tumor infiltration of CD8+ lymphocytes is beneficial, where not just a high overall CD8+ lymphocyte density in the intra-tumoral infiltration region seems to be important but a high proportion of the CD8+ lymphocytes available in the environment infiltrating the tumor as indicated by the feature ratio. The overall densities were also part of the feature set, however, the ratio features yielded the best stratification results. Consequently, even patients with a low lymphocyte density in the TME are likely to be therapy responders if the lymphocyte density in the intra-tumoral infiltration region is about 5 times higher. Thus, our observations agree well with the findings previously published for the MISIPI study claiming that a low CD8 + PD-L1− density in the invasive margin is associated to therapy response^14^. More precisely, we found the same trend in our data when performing median cut point stratification based on CD8+ density in the TME alone (see Supplemental Fig. S5, note, however, that PD-L1 status is not available for our data). The relative density of CD8+ in the whole tumor with respect to CD8+ and CD3+ densities in the surrounding tissue turned out to be likewise predictive with only slightly lower average accuracy and robustness (Table 5, features (4), (5)). This increased infiltration of CD8+ into the tumor might be caused by a high neoantigen burden which has been shown to be present in tumors with strong response to CTLA-4 blockade^9^. Also, as it has been demonstrated by Hamid *et al*.^7^, an increase of TILs during treatment with ipilimumab is related to better therapy response, and a baseline immunosuppressive microenvironment with high expression of T-cell suppressive factors is associated with better outcomes of ipilimumab treatment. This is also consistent with the findings of the MISIPI study which additionally suggests that a high density of CD163+ macrophages may represent a marker for the potential reversibility of this T-cell inhibition upon treatment with ipilimumab^14^.

Interestingly, the observed pattern of low CD8+ densities in the TME and high CD8+ densities at the direct tumoral infiltration front being associated with longer median OS was similarly described for liver metastases in colorectal cancer^32^. While in our case we considered the intra-tumoral infiltration region, and in Berthel *et al*.^32^ the TME in <10 µm distance to the tumor was found to be relevant, both observations might capture the same effect as in our case the tumor border annotation was not highly precise (it was performed on the CD3-stained section and propagated to the CD8-stained section by co-registration) and mostly included the 10 µm distance. Moreover, the combination of the described CD8+ density pattern with an increased CD163+ macrophage density in the TME being associated with prolonged median OS was found for both, malignant melanoma^14^ and colorectal cancer^32^. Closely related to our results, also for anti-PD1 therapy the CD8+ density in the tumor was found to be among the best predictors for therapy response^33^.

To create the best discriminatory model for ipilimumab response prediction we combined the selected best features in a decision tree model, which has been identified as the most appropriate model in a systematic comparison study. In the decision tree generation algorithm the two best features (Tables 5, (1) and (2)) were picked as decision rules at the tree nodes, emphasizing the importance of the relative CD8+ density in the tumor periphery.

The potential heterogeneity of the metastatic disease as well as the low sample number of 31 patients are possible reasons for the moderate average accuracies of 66.3% obtained in the uni-variate case and 70.9% obtained in the multi-variate case for predicting therapy response when using the quite conservative Monte Carlo cross validation procedure. Applying the best feature selection method (10-times 8-fold cross validation) on the whole data set already yields much higher average accuracies of up to 77.4%, where overfitting is avoided by computing the average accuracies on the test folds only. Training and applying the selected multi-variate decision tree model on the whole data yields an overall accuracy of 80.7%, which however, may be biased by overfitting since here no cross validation is involved. Still we can conclude that the multi-variate model is generally superior to the uni-variate models (i.e. accuracy >77.4% on the whole data set) as it has been approved by MCCV. The value of our approach has additionally been demonstrated by using the same framework to obtain a multi-variate model for predicting the duration of survival.

Thus, our results are well in agreement with findings of previous studies and we yield internally validated satisfying accuracies. However, to push forward the development of a clinically relevant approved companion diagnostic test based on our results, additional studies and further validation is required. Most importantly, a larger patient cohort needs to be considered and additional validation on cohorts from different clinical sites should be performed. Nevertheless, the computational methods for data evaluation presented here provide a robust framework for analyzing such validation studies and can be readily applied to any additional data. This also applies to future challenges such as identifying predictive biomarkers for combination therapies. Using the same approaches as developed in this work for combination therapies could help making these promising therapies accessible to the patients profiting most while avoiding unnecessary adverse events caused by inappropriate treatment.

## Supplementary information

Supplementary Material

Software 1

## Data Availability

The datasets generated and analyzed during the current study are currently not publicly available but are available from the authors on reasonable request.
