# Supplementary material for: Automatic discovery of image-based signatures for ipilimumab response prediction in malignant melanoma
Source: Sci Rep. 2019 May 15;9:7449. doi: 10.1038/s41598-019-43525-8 (PMC6520405; doi:10.1038/s41598-019-43525-8)

Automatic discovery of image-based signatures for ipilimumab response prediction in malignant melanoma

N. Harder, R. Schönmeyer, K. Nekolla, A. Meier, N. Brieu, C. Vanegas, G. Madonna, M. Capone, G. Botti, P.A. Ascierto, G. Schmidt

--- Supplemental Material ---

Supplemental Figures

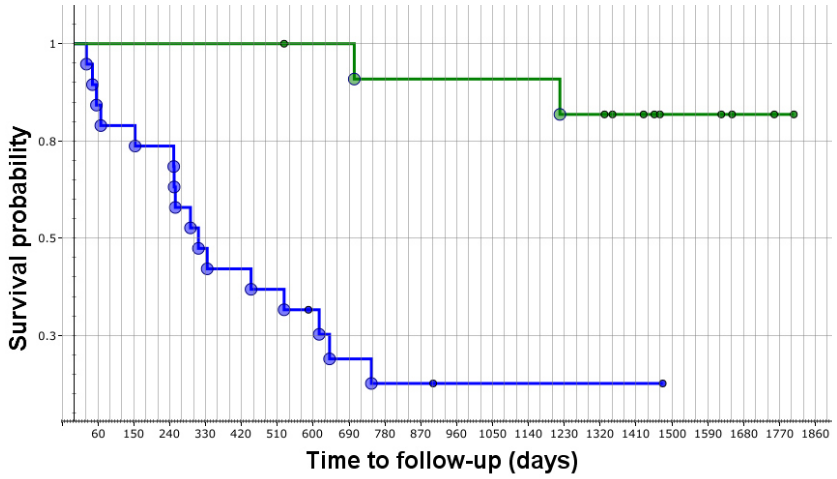

A

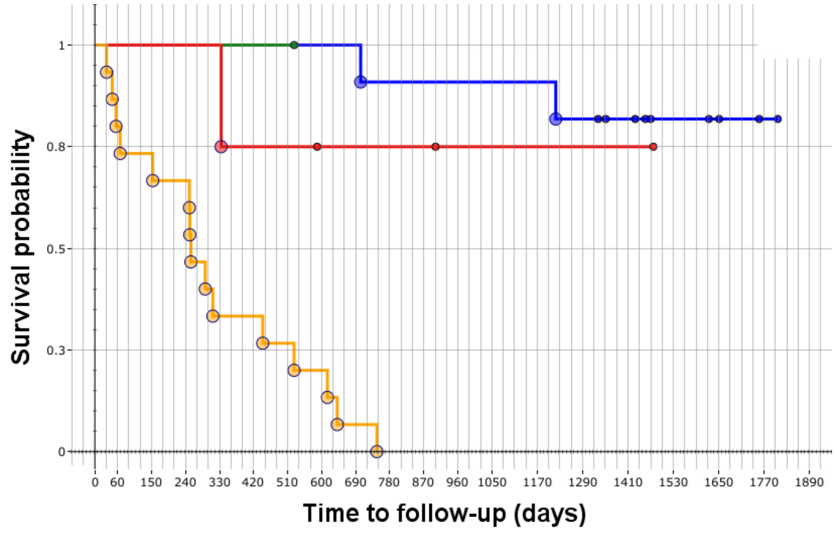

B

Figure S1: Kaplan Meier plots showing the true stratification into (**A**) Ipilimumab responders (green, n=12) versus non-responders (blue, n=19), and (**B**) four different response categories (1) complete response (blue, n=11), (2) partial response (green, n=1), (3) stable disease (red, n=4), and (4) progressing disease (yellow, n=15), where (1) and (2) represent the group of responders, while (3) and (4) represent non-responders.

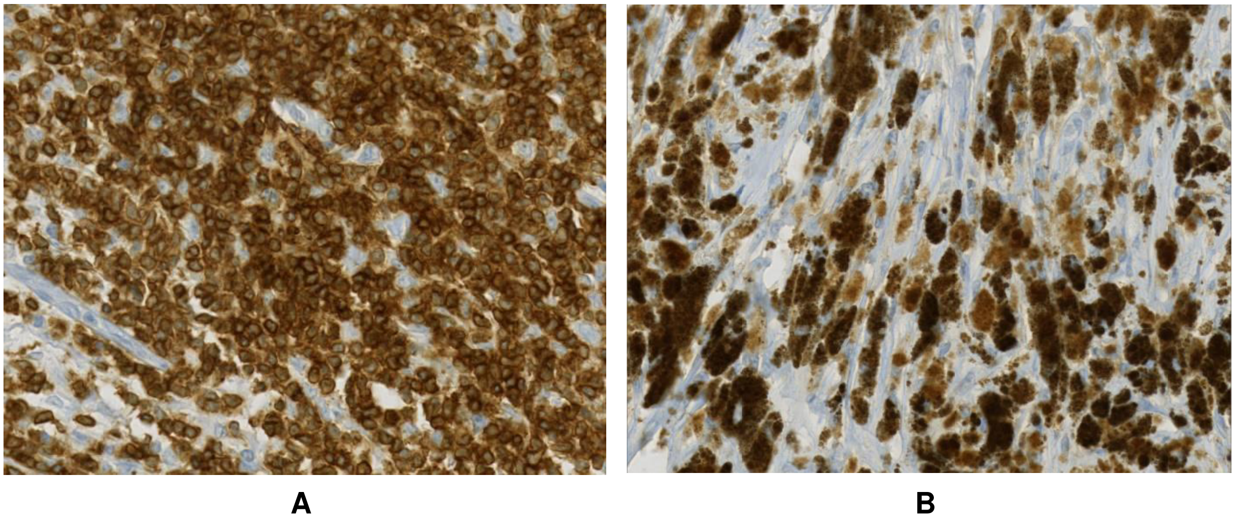

Figure S2: Examples of brown phenotypes: (**A**) CD3+ region, (**B**) melanin region.

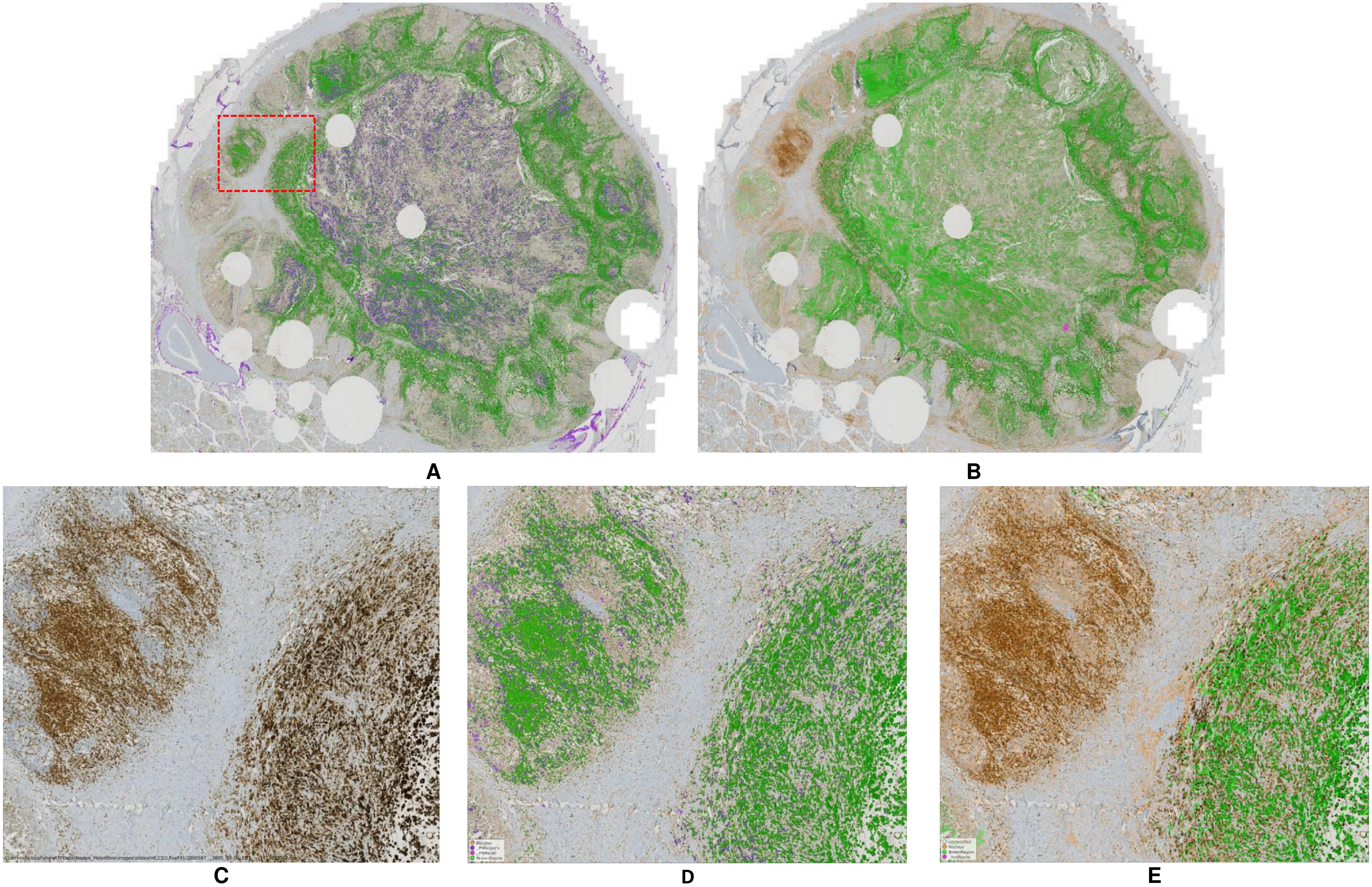

Figure S3: Illustration of automatic patch generation. (**A**) CD3 section with CD3 segmentation overlaid, (**B**) CD3 section with FoxP3 segmentation overlaid, (**C**) zoomed region (red dashed box in **A**), raw CD3 image with CD3+ region top-left and melanin region bottom-right, (**D**) zoomed region CD3 segmentation, (**E**) zoomed region FoxP3 segmentation. Overlays: (green) IHC-positive, (orange) IHC-negative, (blue) artifact.

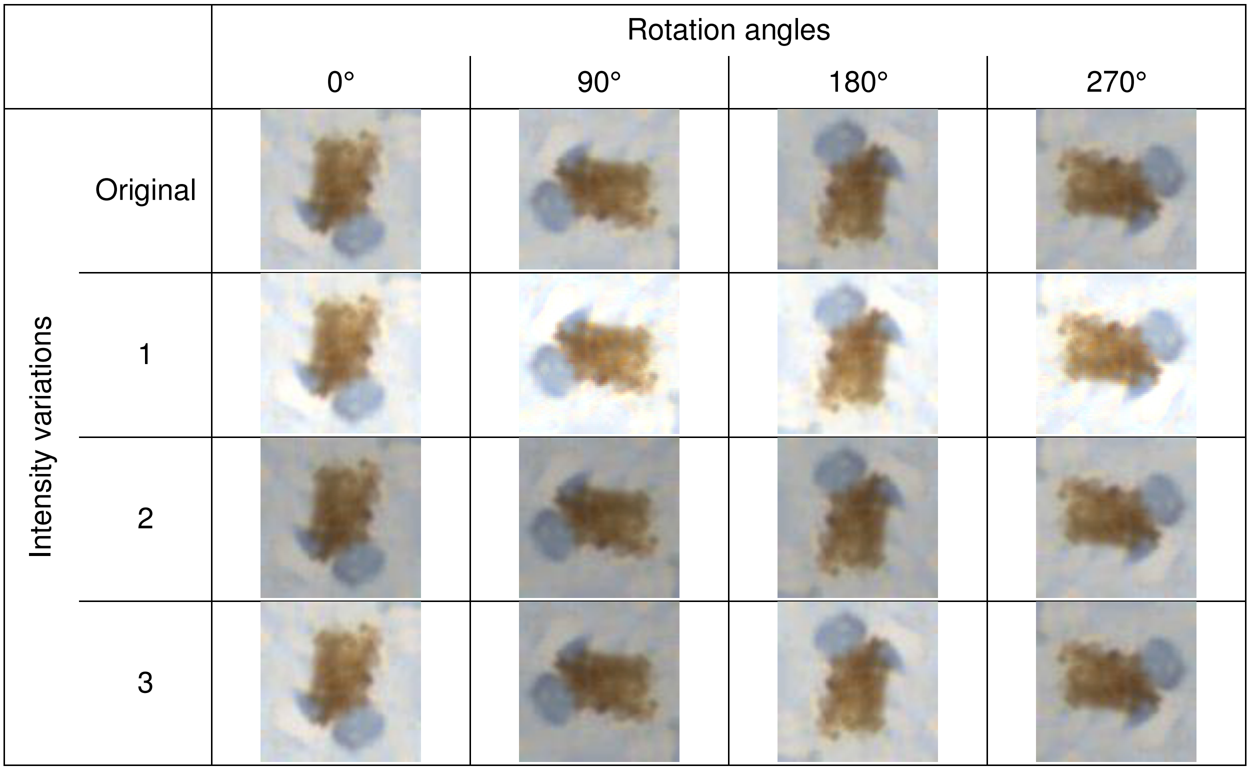

Figure S4: Example data augmentation for a patch of class non-specific stain (original patch: top left corner)

Feature	Box plot<br>Non-response Response	(A) Kaplan-Meier plot with **median** cut-off	(B) Kaplan-Meier plot with **optimized** cut-off
(1)<br>CD8+ density in TME small	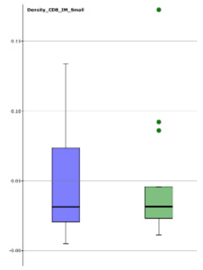	Value > median Value <= median<br>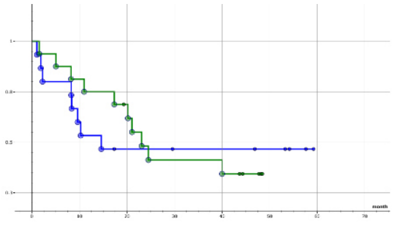	Value > 0.034 Value <= 0.034<br>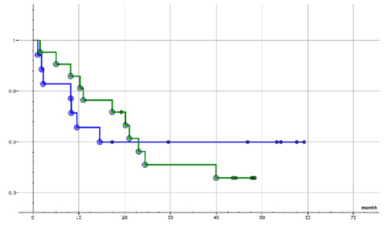
(2)<br>CD8+ density in TME large	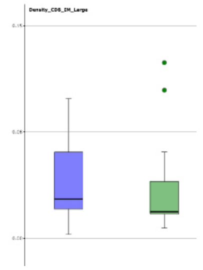	Value > median Value <= median<br>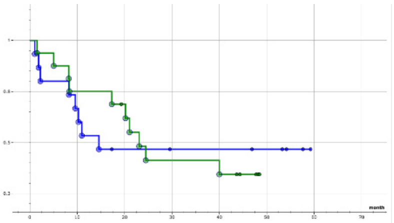	Value > 0.020 Value <= 0.020<br>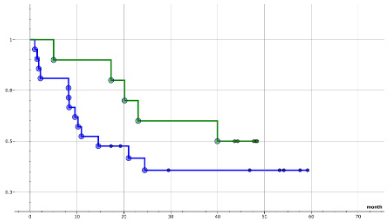
(3)<br>CD8+ density in stroma	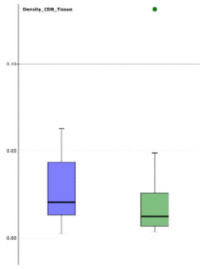	Value > median Value <= median<br>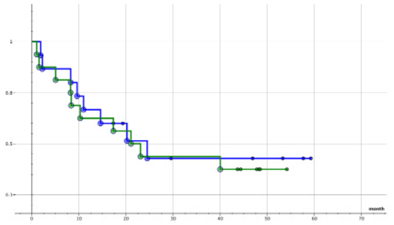	Value > 0.013 Value <= 0.013<br>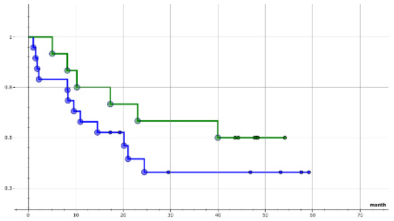
(4)<br>CD8+ density in intra-tumoral infiltration / CD8+ density in stroma	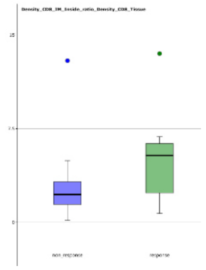	Value > median Value <= median<br>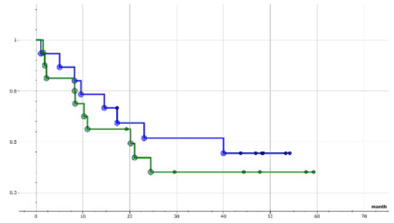	Value > 5.2 Value <= 5.2<br>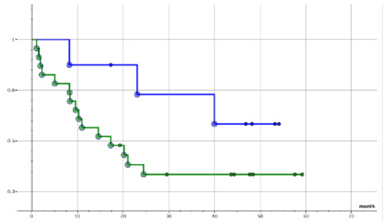
(5)<br>CD8+ density in intra-tumoral infiltration	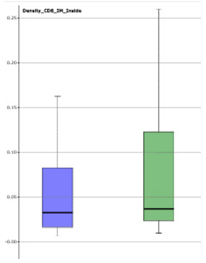	Value > median Value <= median<br>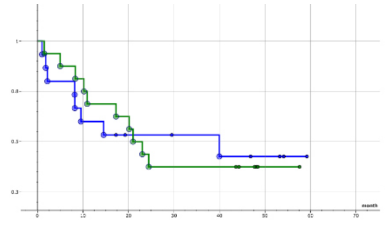	Value > 0.05 Value <= 0.05<br>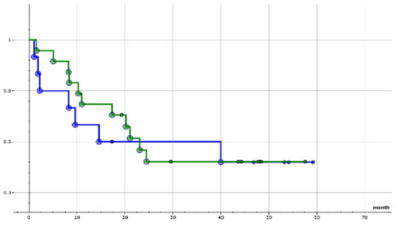

Figure S5: Box plots and Kaplan-Meier plots for basic cut point analysis without cross validation for selected features for IPI response prediction. (A) Median feature values as cut points, (B) optimized cut points.

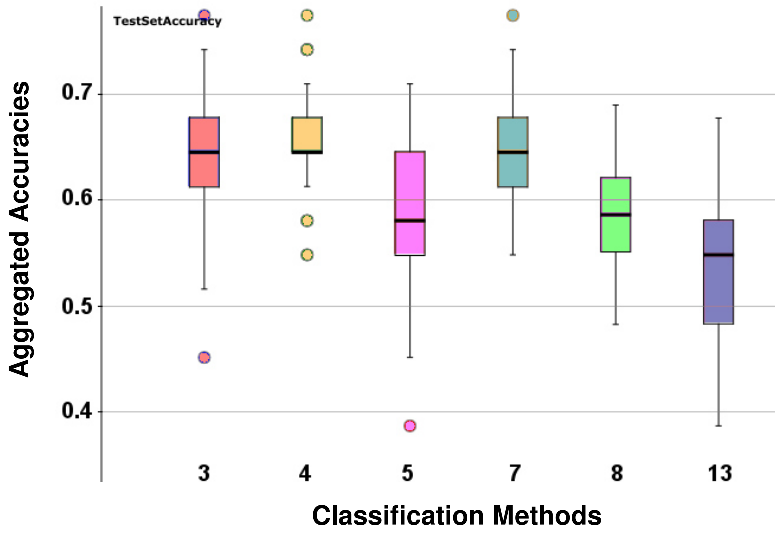

Figure S6: Evaluation of multi-variate models using the Monte Carlo cross validation (M=50) regarding their performance for predicting the duration of survival (*short-term*: <=12 months, *long-term*: >12 months). Only the best-performing models are included (see main manuscript **Figure 11**): (3) decision tree (d=2, s_{min}=5, l_{min}=3), (4) decision tree (d=2, s_{min}=5, l_{min}=5), (5) decision tree (d=2, s_{min}=5, l_{min}=5), information gain measure: *cross entropy*, (7) decision tree (d=3, s_{min}=5, l_{min}=3), (13) logistic regression, 5 features.

Supplemental Tables

T-test	2	3	4	5	6
1	0.9514	0.0008257	3.64e-21	3.69e-16	9.48e-08
2		0.002238	4.69e-19	1.20e-14	1.48e-07
3			5.43e-13	9.68e-09	7.62e-12
4				0.09382	4.50e-22
5					5.80e-20
MW-test	2	3	4	5	6
1	0.7493	0.0002436	1.97e-15	2.47e-13	1.93e-07
2		0.001721	7.57e-15	1.91e-12	2.42e-07
3			2.04e-11	2.96e-08	8.63e-11
4				0.1064	4.95e-16
5					3.87e-15

Table S1: Statistical tests for comparison of feature selection methods for IPI response prediction. Top: Two-sided t-test, bottom: Mann-Whitney test. The p-values in the shaded cells correspond to the finally selected best approach (4) 10-times 8-fold cross validation.

T-test	2	3	4	5	6	7	8	9	10	11	12	13	14	15
1	5.82e-07	0.02154	4.64e-06	0.00019	0.5283	0.3943	0.1152	1.23e-06	1.55e-15	8.54e-12	6.29e-14	0.02165	7.13e-10	1.41e-06
2		1.14e-13	8.78e-20	2.53e-17	4.26e-07	6.40e-05	0.00106	0.3288	2.00e-08	0.00044	2.91e-06	0.00258	0.04066	0.8734
3			0.00630	0.08714	0.00113	0.00210	0.00015	3.80e-11	1.80e-19	3.55e-17	9.89e-19	2.08e-06	1.98e-16	5.06e-13
4				0.298	1.31e-08	2.32e-07	6.99e-09	1.28e-15	5.38e-23	8.40e-22	7.31e-23	1.04e-11	5.49e-22	4.71e-19
5					1.69e-06	1.10e-05	4.33e-07	7.14e-14	1.13e-21	5.18e-20	2.76e-21	1.55e-09	8.78e-20	1.24e-16
6						0.737	0.2549	2.30e-06	3.01e-15	7.59e-12	7.23e-14	0.05379	3.45e-10	1.29e-06
7							0.4794	4.50e-05	8.66e-14	1.07e-09	6.41e-12	0.1811	1.33e-07	0.00013
8								0.00047	1.47e-12	2.45e-08	1.40e-10	0.5649	3.31e-06	0.00185
9									9.22e-06	0.03261	0.00089	0.00119	0.4876	0.2775
10										0.00563	0.1428	2.78e-12	1.73e-05	1.48e-08
11											0.1644	4.06e-08	0.08333	0.00033
12												2.20e-10	0.00203	2.16e-06
13													5.69e-06	0.00467
14														0.03106

MW-test	2	3	4	5	6	7	8	9	10	11	12	13	14	15
1	3.06e-06	0.03376	2.04e-05	0.00041	0.6267	0.4938	0.1672	8.05e-06	3.25e-13	4.09e-10	1.79e-11	0.05483	1.36e-08	6.17e-06
2		2.58e-11	1.55e-14	1.92e-13	1.02e-06	7.05e-05	0.00081	0.4581	7.77e-08	0.00058	1.57e-05	0.00138	0.03665	0.9206
3			0.00692	0.07626	0.00192	0.00590	0.00027	8.55e-10	8.60e-16	8.70e-14	8.74e-15	6.86e-06	7.86e-13	4.60e-11
4				0.3141	9.39e-08	9.97e-07	4.37e-08	4.66e-13	5.81e-17	9.84e-16	2.22e-16	2.80e-10	2.77e-15	3.62e-14
5					3.71e-06	2.93e-05	1.30e-06	6.63e-12	1.50e-16	6.15e-15	8.88e-16	1.16e-08	2.04e-14	4.53e-13
6						0.9354	0.3048	1.21e-05	1.30e-13	2.97e-10	8.33e-12	0.107	5.68e-09	1.91e-06
7							0.408	8.09e-05	6.76e-12	6.48e-09	2.88e-10	0.14	3.96e-07	9.78e-05
8								0.00092	4.23e-11	1.12e-07	2.99e-09	0.6139	5.15e-06	0.00107
9									2.94e-05	0.03552	0.00163	0.00239	0.3929	0.4195
10										0.01356	0.2233	4.37e-11	6.91e-05	6.42e-08
11											0.1999	2.89e-07	0.1135	0.00057
12												4.42e-09	0.00570	1.31e-05
13													9.83e-06	0.00173
14														0.03075

Table S2: Statistical tests for comparison of multi-variate models for IPI response prediction. Top: Two-sided t-test, bottom: Mann-Whitney test. The p-values in the shaded cells correspond to the finally selected best approach (4) CART (depth=2, $split_{min}$=5, $leaf_{min}$=5, information gain=gini).

T-test	4	5	7	8	13
3	0.0131	0.0002	0.0560	2.77e-05	4.33e-11
4		7.27e-09	0.4128	3.04e-11	1.23e-16
5			4.29e-08	0.9347	0.0013
7				1.22e-10	6.46e-16
8					0.0002
MW-test	4	5	7	8	13
3	0.0232	0.0002	0.1160	0.0002	5.82e-10
4		5.78e-08	0.3641	8.59e-09	1.81e-13
5			2.33e-07	0.3372	0.0022
7				3.44e-08	2.20e-13
8					2.95e-05

Table S3: Statistical tests for comparison of different multi-variate models for predicting the duration of survival. Top: Two-sided t-test, bottom: Mann-Whitney test. (3) decision tree (d=2, s_{min}=5, l_{min}=3), (4) decision tree (d=2, s_{min}=5, l_{min}=5), (5) decision tree (d=2, s_{min}=5, l_{min}=5), information gain measure: *cross entropy*, (7) decision tree (d=3, s_{min}=5, l_{min}=3), (13) logistic regression, 5 features.

Supplemental Code

Flow Chart Symbols

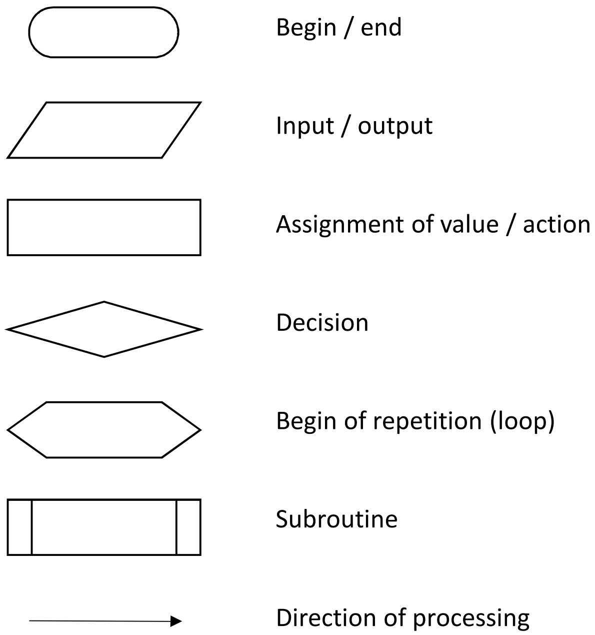

References

[1] Brieu, N., Pauly, O., Zimmermann, J., Binnig, G., Schmidt, G. “Slide specific models for segmentation of differently stained digital histopathology whole slide images”, in *SPIE Med. Imaging* 2016.

[2] Yigitsoy, M. & Schmidt, G. “Hierarchical patch-based co-registration of differently stained histopathology slides”, in *SPIE Med. Imaging* 2017.

[3] Harder, N., Athelogou, M., Hessel, H., Brieu, N., Yigitsoy, M., Zimmermann, J., Baatz, M., Buchner, A., Stief, C. G., Kirchner, T., Binnig, G., Schmidt, G., Huss, R. “Tissue Phenomics for prognostic biomarker discovery in low- and intermediate-risk prostate cancer”, *Sci. Rep. 8*, 4470, 2018.

For pseudo codes see Supplemental Material.

2.5.1. Automatic training data generation

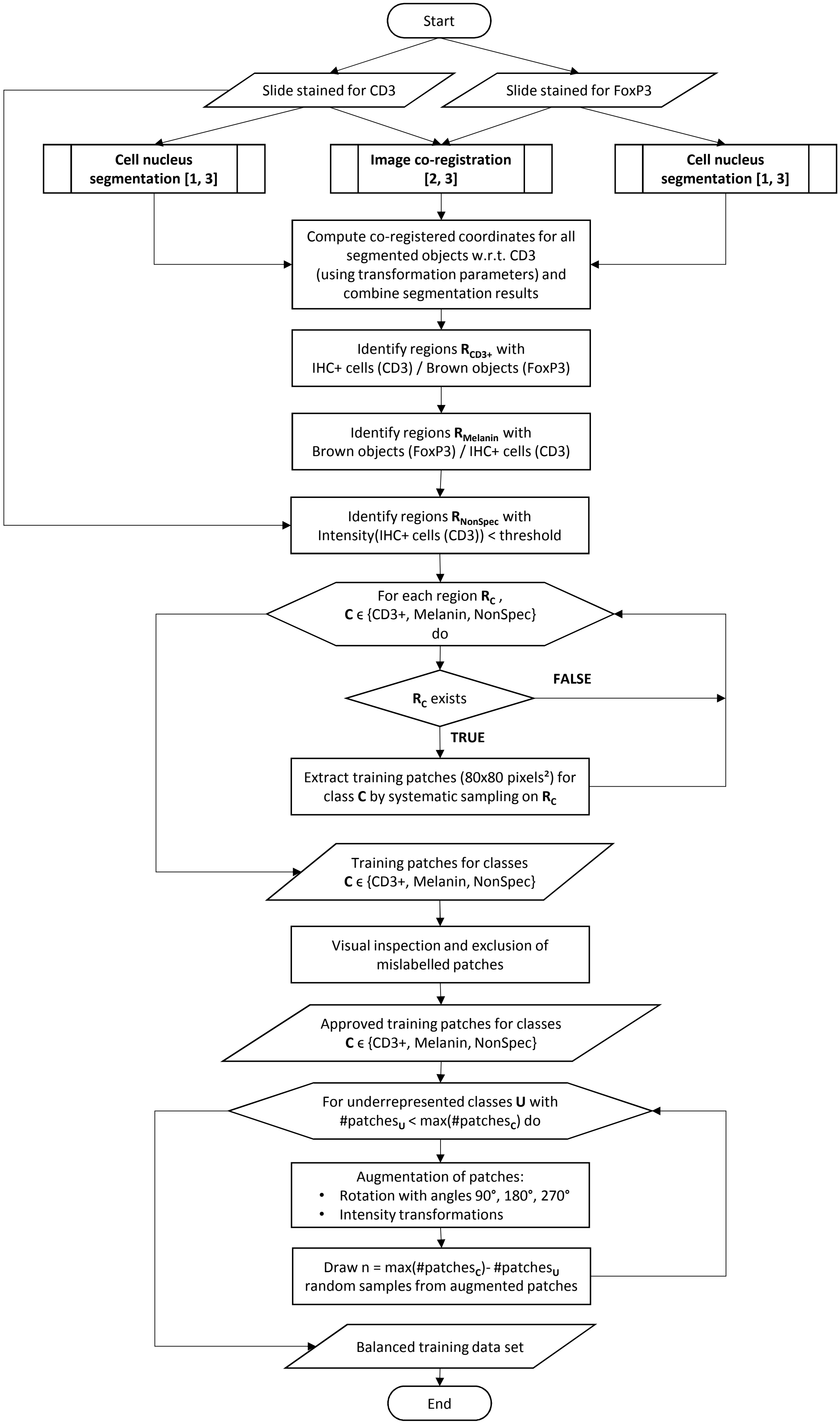

Supplement: Supplementary file 1 — Supplementary Material [file 41598_2019_43525_MOESM1_ESM.pdf]
